# Exploration of immune correlates of viral clearance after PVX2 immunotherapy in patients with human papillomavirus type 16 infection and cervical intraepithelial neoplasia grade ≤1

**DOI:** 10.1128/jvi.00643-26

**Published:** 2026-07-27

**Authors:** Vikrant Palande, Tianbei Zhang, Kellie N. Smith, Kimberly Levinson, Leslie Cope, Hua-Ling Tsai, Yung-Nien Chang, T.-C. Wu, Mark H. Einstein, Richard B. S. Roden

**Affiliations:** 1 Department of Pathology, Johns Hopkins University School of Medicine 1500, Baltimore, Maryland, USA; 2 Department of Oncology, Johns Hopkins University School of Medicine 1500, Baltimore, Maryland, USA; 3 Department of Gynecology/Obstetrics, Johns Hopkins University School of Medicine 1500, Baltimore, Maryland, USA; 4 Department of Biostatistics, Johns Hopkins University School of Medicine 1500, Baltimore, Maryland, USA; 5 PapiVax Biotech Incorporated, Taipei, Taiwan; 6 Department of Obstetrics & Gynecology and Women's Health, Montefiore Medical Center and Albert Einstein Comprehensive Cancer Center 733378, Bronx, New York, USA; Tufts University, Boston, Massachusetts, USA

**Keywords:** HPV, immunotherapy, CIN, LSIL, cervical cancer

## Abstract

Immune responses correlated with clearance of human papillomavirus type 16 (HPV16) were sought in PVX2 immunotherapy-treated CIN ≤1 patients. PVX2 comprises intramuscular vaccinations with HPV16 E7-targeted pNGVL4a-Sig/E7(detox)/HSP70 DNA vaccine (3 mg, weeks 0 and 4) and HPV16 L2E7E6 fusion protein (TA-CIN; 100 µg week 8). Administering PVX2 to 12 women with HPV16+ CIN ≤1 elicited both antibody and CD8+ T-cell responses, including changes in TCR diversity, clonal expansion, and emergence of public clonotypes. HPV16 was not detected in 5/11 evaluable participants at month 6. Post-vaccination E6, E7, and L2-specific antibody titers were higher, but none correlated with clearance. Neither TCR clonal diversity/expansion nor public clonotypes were associated with HPV16 clearance. While the fractions of expanded/new clonotypes were not associated with HPV16 clearance, a higher proportion of vaccine antigen-specific reads was evident post-vaccination in those clearing HPV16. High baseline serum MCP-1 associated with HPV16 persistence, and MIP-1β trended higher post-vaccination in those clearing HPV16.

**IMPORTANCE:**

HPV16 is a necessary cause of over half of all cervical cancers, and in even higher fractions of other anogenital and oropharyngeal cancers. Treatments for HPV16 infection and associated pre-cancerous lesions are needed given the disease burden and limitations of current treatments. Women with persistent HPV infections endure anxiety and stress due to frequent colposcopies, inconvenience, healthcare costs from active surveillance, and potential to transmit the virus to other sexual partners. Since a majority of patients can spontaneously clear their infections, administration of an immunotherapy may promote the elimination of otherwise persistent infections where disease can progress.

## INTRODUCTION

Persistent infection of the cervical squamous epithelium with a high-risk (hr) HPV type is a necessary but insufficient cause of cervical cancer (1). HPV type 16 alone accounts for over half of all cases of cervical malignancies (2). HPV16 infects the genital mucosa and initially forms a productive lesion termed cervical intraepithelial neoplasia (CIN) grade 1 (3). In patients with an intact immune system, most genital hrHPV infections clear spontaneously without sequelae (4). HPV16, however, is the most persistent of all genital high-risk types, and for persistent infections (5), HPV16 is the type that most frequently progresses to high-grade dysplasia (CIN2/3), the precursor of cervical cancer (6, 7). In a large cohort of older women (mean age 38.5 years), HPV16 infections associated with borderline or mild dyskaryosis showed low clearance rates 6 months later of 9% (95% CI 5–17), and only 19% (95% CI 12–29) by 18 months (5). While HPV16+ CIN2/3 has low rates of spontaneous clearance (8, 9), HPV16 infections in women with normal cytology showed much higher clearance rates at 6 months of 34% (95% CI 26–42), and 49% (95% CI 41–59) at 18 months. Higher rates of spontaneous HPV16 clearance are reported in younger populations, e.g., 45% (95% CI, 40–50) in 12 months in participants aged 18–25 years with benign cytology (10).

To prevent cervical cancer, CIN2/3 is removed with cold knife conization (CKC) or a loop electrosurgical excision procedure (LEEP) (11, 12). Unfortunately, these ablative treatments are associated with significant side effects such as bleeding, cervical stenosis, and pre-term delivery, and do not target the virus (11, 12). Since HPV testing is becoming widely used in cervical screening, a virus-targeted treatment for HPV16 infection and low-grade disease could provide additional opportunity for cervical cancer prevention. Effective treatment of a persistent infection holds the promise of avoiding the need for CKC/LEEP as well as the other sequelae of frequent surveillance (13). High-grade vaginal, vulvar, and anal intraepithelial lesions are most frequently driven by HPV16. Their surgical resection is associated with high recurrence rates, in part because it does not target the underlying cause, HPV infection (14, 15).

Rates of HPV-associated cancers are higher, and clearance rates of HPV infection are lower, in HIV+ patients and organ transplant recipients on immunosuppressive regimens, suggesting the importance of cellular immunity in viral control (16). Immunotherapies targeting appropriate HPV16 antigens have the potential to elicit cytotoxic T-cell responses able to control productive HPV infections (17). Studies in animal models suggest that immunotherapeutic strategies targeting the viral oncoproteins E6 and/or E7 can induce viral clearance and HPV16+ tumor control (18–20). By contrast, vaccination against minor capsid antigen L2 induces neutralizing antibodies that protect animals from infection, and L2 is generally considered a prophylactic antigen only (21–24). However, in some models, L2 vaccination does promote disease clearance (25, 26).

The naked DNA vaccine pNGVL4a-Sig/E7(detox)/HSP70, which expresses a secreted fusion of HPV16 E7 linked to the *Mycobacterium tuberculosis* heat shock protein 70 (HSP70) molecular adjuvant (27), elicits potent E7-specific CD8 T-cell immune responses that control HPV16+ tumor in mice (19). Repeat intramuscular pNGVL4a-Sig/E7(detox)/HSP70 vaccination in women with HPV16+ CIN2/3 is well tolerated at 3.0 mg, with 3/9 subjects exhibiting a complete histologic regression of disease (19).

Studies in models of malaria (28) and HIV (29–31) suggest that administration of a recombinant protein booster following a priming DNA vaccination is a promising approach to further enhance the T-cell response and better control an infectious agent. Intramuscular administration of pNGVL4a-Sig/E7(detox)/HSP70 DNA vaccine twice followed by boosting with a recombinant HPV16 L2E7E6 fusion protein (termed TA-CIN [32, 33]) generated significantly more HPV-specific CD8+ T cells in mice as compared to either DNA vaccination or protein-based vaccine alone (34), and improved control of the HPV16+ TC-1 tumor model. In a single-arm study (NCT03911076), the PVX2 regimen, comprising two 3 mg pNGVL4a-Sig/E7(detox)/HSP70 DNA and one 100 µg TA-CIN intramuscular vaccination administered 1 month apart, was well tolerated in twelve patients (mean age 43.5 years) with HPV16 infection and CIN ≤1 (35). Eleven participants returned for HPV testing at the primary endpoint (month 6), and five had no detectable HPV16 (45%). All five responders remained HPV16 negative at month 12. Two of the six non-responder participants that failed to clear HPV16 developed CIN2/3 and underwent a LEEP procedure. For context, the average age of the Phase I study participants and their grade of HPV16+ disease is similar to a large natural history cohort for which a clearance rate of 9% (95% CI 5–17) at 6 months was observed (5). However, since the PVX2 study was uncontrolled, it is not clear whether the clearance of HPV16 was spontaneous or elicited by the PVX2 immunotherapy.

Here, we examine whether the DNA and TA-CIN vaccinations were associated with HPV16 E6, E7, or L2-specific CD8+ T-cell and antibody responses detectable in peripheral blood samples of the PVX2 study participants, and explore whether these responses to PVX2 correlate with virologic clearance. Serum cytokine levels were similarly assessed.

## MATERIALS AND METHODS

### Study design and patient population

Samples from the safety run-in cohort of a previously reported clinical trial (NCT03911076) evaluating the PVX2 therapeutic HPV vaccine in women with HPV16-positive low-grade cervical lesions were analyzed (35).

### HPV16 E6, E7, and L2 antigen ELISA

6His-tagged HPV16 E6, E7, and L2 were expressed in *E. coli* and purified under denaturing conditions according to the QiaExpressionist manual. Direct ELISAs were performed as described in the supplemental methods.

### Multiplex cytokine bioplex assays

A multiplex cytokine bead array kit (Bio-Rad) was used to simultaneously quantify 17 total cytokines and chemokines in human serum according to the manufacturer’s protocol.

### Analysis of peripheral blood T-cell repertoires by T-cell receptor sequencing (TCRseq)

T-cell receptor beta chain (TCRβ) repertoires were profiled from genomic DNA extracted from cryopreserved PBMCs using the QIAamp DNA Micro Kit (QIAGEN, catalog #56304) according to the manufacturer’s instructions. TCRβ libraries were prepared and sequenced by the Johns Hopkins FEST and TCR Immunogenomics Core Facility (FTIC) using the AmpliSeq for Illumina TCR beta-SR (Short Read) panel, which employs multiplex PCR with primers targeting all functional TRBV and TRBJ gene segments to amplify the CDR3 region, which is the hypervariable loop that determines antigen specificity. T-cell receptor (TCR) sequencing data were analyzed using R. For each sample, TCR sequences were first aggregated by their CDR3 amino acid sequences, with frequencies calculated as the proportion of total reads per sample. Repertoire diversity was quantified using four complementary indices: Shannon diversity, Inverse Simpson index, Chao1 estimator, and D50 index. Clonality was calculated using the inverse normalized Shannon entropy: 1 − (−∑(pi × log2(pi))/log2(N)), where pi represents the frequency of clone i, and N is the total number of unique clones (36). To identify significantly expanded T-cell clones within uncultured peripheral blood T cells, Fisher’s exact test was performed for each clone by comparing the frequency between pre- and post-vaccination time points. *P*-values were adjusted for multiple testing using the Benjamini-Hochberg method. Clones were classified as significantly expanded if they met three criteria: (i) adjusted *P*-value < 0.05, (ii) higher frequency in post-vaccination compared with pre-vaccination samples, and (iii) a minimum frequency threshold of 0.01%. Expanded clones were further categorized as either “*de novo*” (detected only post-vaccination) or “pre-existing” (present before, but at a higher frequency after vaccination). Three distinct metrics were computed to characterize the TCR repertoire response: (i) clonality, which measures the overall diversity of the repertoire; (ii) proportion of expanded clones, calculated as the number of significantly expanded clones divided by the total number of clones; and (iii) clonal space, defined as the sum of frequencies of expanded clones. These metrics were calculated separately for *de novo* expanded clones and pre-existing expanded clones. Statistical comparisons between the responders and non-responders were performed using two-sided Wilcoxon rank-sum tests, with *P*-values < 0.05 considered statistically significant. More details are supplied in the supplemental methods.

### Identification of HPV-specific T cells utilizing MANAFEST

Overlapping peptide pools spanning the HPV16 E6, E7 and L2 (JPT Peptide Technologies, Berlin, Germany) were used to stimulate CD8^+^ T cells in the MANAFEST assay, as described previously (37, 38), with minor modifications. Briefly, 2 × 10^6^ PBMCs were plated in culture medium (IMDM, 5% human AB serum, 10 IU/mL IL-2, 50 μg/mL gentamicin) with 5 μg/mL of individual HPV E6, E7, and L2 peptide pools, a positive control CEFX Ultra SuperStim consisting of pooled CMV/EBV/Influenza (CEF) MHC I-restricted epitopes (JPT), or without peptide. Each assay condition was in triplicate when the cell number allowed. On day 3, half of the culture medium was replaced with fresh medium containing IL-2 (100 IU/mL), IL-7 (50 ng/mL), and IL-15 (50 ng/mL). On Day 7, a second half-medium exchange was performed with fresh medium containing IL-2 (200 IU/mL), IL-7 (50 ng/mL), and IL-15 (50 ng/mL). On day 10, the cells were harvested and CD8^+^ T cells were isolated using the EasySep CD8^+^ T-cell isolation kit (STEMCELL, 17952). DNA was extracted from cultured CD8^+^ T cells using the QIAamp Micro-DNA Kit, according to the manufacturer’s instructions (QIAGEN). T-cell receptor (TCR) sequencing of DNA extracted from cultured CD8^+^ T cells was performed by the Johns Hopkins FEST and TCR Immunogenomics Core Facility (FTIC) using the AmpliSeq for the Illumina TCR beta-SR panel. The samples were pooled and sequenced using Illumina MiSeq. Data preprocessing was performed to eliminate non-productive TCR sequences, and to align and trim the nucleotide sequences to obtain only the CDR3 region. Sequences not beginning with C, ending with F or W, and having fewer than seven amino acids in CDR3 were eliminated. The resultant processed data files were imported using the *repload* package from the immunarch R package (39). Sequences supported by <50 total reads across all peptide pools were excluded from further analyses. Multivariate negative binomial regression was used to model the count data and identify clonal expansions in individual peptides, which are represented in the model as interactions between clones and peptides. Each sequence was modeled independently, in comparison to the pool of all other clones, with all peptides included in the same multivariate model along with the vehicle control as the model intercept. Expansion was reported as an odds ratio, together with a 95% CI and *P*-value. To select the highest quality sequences, those with OR > 1 were considered significant if after multiple test corrections, the Benjamini-Hochberg false discovery rate (FDR) was <0.05. To identify clones uniquely responsive to a single antigen, a uniqueness filter was applied: clones were required to show statistically significant enrichment in only one peptide condition (the “best” condition) and to be significantly different from both the second-best and third-best conditions. This was implemented by comparing the best-responding condition to the second-best and third-best conditions using additional statistical tests, requiring OR ≥ 1 and FDR < 0.05 for both comparisons. Further details are provided in the supplemental methods.

## RESULTS

The study cohort comprised peripheral blood samples (PBMC and sera) from 12 female participants (median age 39 years, range: 29–58 years, 11 White and 1 Asian, with 5 patients (42%) identifying as Hispanic or Latino). Participants entered the study with HPV16 infection and low-grade squamous intraepithelial lesion (LSIL/CIN1), atypical squamous cells of undetermined significance (ASC-US), or atypical squamous cells cannot exclude high-grade lesion (ASC-H). All received two 3 mg doses of pNGVL4a-Sig/E7(detox)/HSP70 DNA plasmid (Weeks 0 and 4), followed by one dose of the HPV16 L2E7E6 fusion protein TA-CIN (week 8). All patients completed the study except one who was lost to follow-up after week 12. The primary clinical endpoint was HPV16 clearance in Month six cytology samples, defined by negative HPV16 testing using the HPV16 18/45 Aptima Assay. Patients were classified as responders (HPV16 cleared at month 6, *n* = 5) or non-responders (HPV16 persistent, *n* = 6) based on this endpoint. Blood samples were collected at weeks 0, 4, 8, 12 and at follow-up visits (month 6 and month 12) and processed for serum and viable PBMC.

### Antibody responses to HPV16 antigens

#### Antibody responses after vaccination

Direct ELISA was used to assess HPV16-specific antibody responses in patient sera. All 11 patients with available baseline samples showed detectable serum IgG titers against HPV16 E6, E7, and L2 proteins at Week 0. EC₅₀ curves demonstrated excellent fit quality, with 100% model convergence and nearly all R² values exceeding 0.98 (minimum R² = 0.976). Median baseline EC₅₀ values were 46.0 for E6, 65.8 for E7, and 62.0 for L2 (Fig. S1).

**Fig 1:**
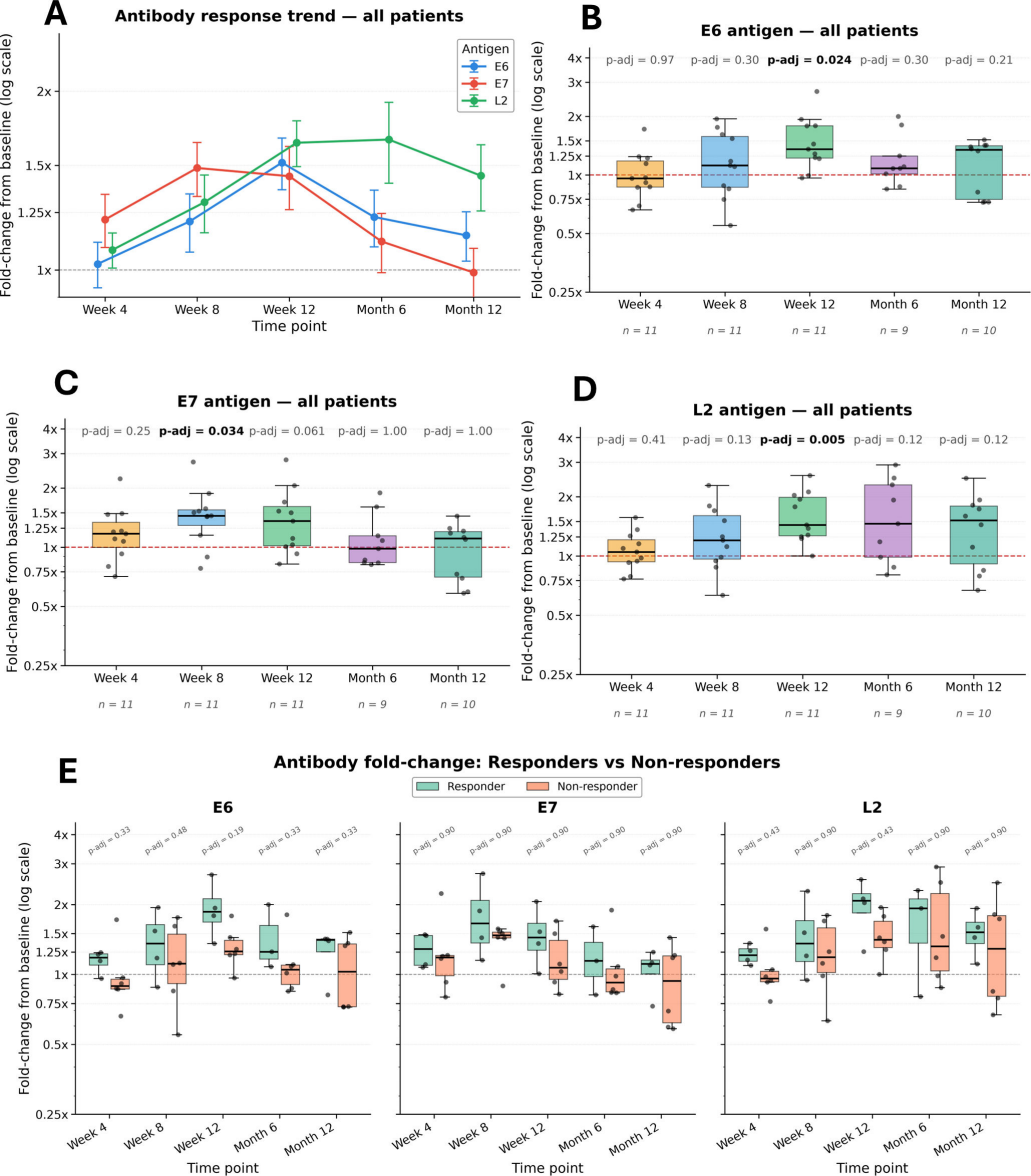
Vaccination induces HPV16-specific antibody responses and a responder-associated anti-E6 fold-increase. (**A**) Longitudinal fold-change in serum IgG antibody titers (relative to baseline week 0) against HPV16 E6 (blue), E7 (red), and L2 (green) proteins, showing mean ± SE across all patients. Dashed line indicates no change from baseline (1×). E6 and L2 showed peak responses at week 12, while E7 peaked at week 8. (**B**–**D**) Fold-change at each timepoint for individual antigens: (**B**) E6, (**C**) E7, and (**D**) L2. Box plots show median with interquartile range; individual patient data points are overlaid. Statistical significance was assessed by comparing fold-change to 1.0 at each timepoint and Mann-Whitney U test with Benjamini-Hochberg correction. For panels **A**–**D**: *n* = 11 patients through week 12 (one patient excluded due to missing baseline sample); *n* = 9 at month 6 (one additional patient lost to follow-up, one missing sample); *n* = 10 at month 12 (one patient lost to follow-up). (**E**) Comparison of antibody fold-change (relative to baseline) between responders (HPV16 cleared at month 6, *n* = 4) and non-responders (HPV16 persistent, *n* = 6) across all timepoints for E6, E7, and L2 antigens; dashed horizontal line indicates no change from baseline (1×); Mann-Whitney U test with Benjamini-Hochberg correction was used to compare response groups at each timepoint. Panel E excludes one responder (1003-009) due to missing baseline sample; *n* = 3 responders at month 6 (one missing sample).

To assess whether these signals represented true antigen-specific binding rather than non-specific background, we compared patient EC₅₀ values with a no-antigen control (week 0 serum tested against buffer-coated wells lacking HPV16 antigen). Patient EC₅₀ values were significantly higher than control at all timepoints, including baseline (mean 68.4 vs 39.4, ratio 1.73×, *P* = 0.025, Mann-Whitney U test; Fig. S2). While this suggests HPV antigen-specific antibody detection, it does not rule out reactivity with contaminants of the HPV antigen. This difference increased following vaccination, peaking at week 12 (ratio 2.83×, *P* < 0.001). Vaccination induced weak but significant increases in antibody titers against all three HPV16 antigens (Fig. 1). Significant E7-specific IgG responses were observed at week 8 (median 1.45-fold, *P*-adj = 0.034, Mann-Whitney U test with Benjamini-Hochberg correction) (Fig. 1C), corresponding to 1 month after the second dose of the E7 targeted DNA vaccine, consistent with preclinical studies (34). No significant change in E6 or L2-specific IgG titer from baseline was detected at this time point, as expected. A weak E6-specific IgG response (median 1.36-fold increase from baseline at week 12, *P*-adj = 0.024, Mann-Whitney U test with Benjamini-Hochberg correction) was evident one month after administration of TA-CIN (Fig. 1B). Likewise, an L2-specific IgG response was detected at Week 12 (median 1.44-fold, *P*-adj = 0.005, Mann-Whitney U test with Benjamini-Hochberg correction). Although TA-CIN contains all three antigens, no further increase in E7-specific IgG was evident at week 12 (Fig. 1C). The E6, E7, and L2-specific IgG responses waned (non-significant by month 6), although only the L2-specific trends toward persistence at month 6 (median 1.46-fold, *P*-adj = 0.12) and also month 12 (median 1.52-fold, *P*-adj = 0.12) (Fig. 1D). Peak fold-change responses for E6 and L2 occurred a month after TA-CIN administration, while for E7 they peaked a month after the second DNA vaccination. Responses declined by Month six but remained elevated above baseline through Month 12 for L2 only.

#### Anti-E6 IgG responses associate with HPV16 clearance

To identify potential immune correlates of clinical response, we compared antibody fold-changes between responders (HPV16 cleared at Month 6, *n* = 4 with baseline samples) and non-responders (HPV16 persistent, *n* = 6) using Mann-Whitney U tests with Benjamini-Hochberg correction. Responders showed a trend for greater fold-increases in anti-E6 antibody titers compared to non-responders at week 12 only (median 1.86-fold vs 1.26-fold, *P*-adj = 0.19; Fig. 1E), whereas there were no clear differences in E7 and L2 fold-change in titers at any timepoint.

### Serum cytokine responses

#### Cytokine profiles during vaccination

Serum concentrations of 17 cytokines and chemokines were measured across all timepoints to characterize systemic immune activation during vaccination. Cytokine levels varied substantially between patients, reflecting individual baseline immunological states. Several cytokines, including IL-4, showed concentrations below the assay detection limit for most samples and were excluded from comparative analyses. MCP-1 (monocyte chemoattractant protein-1/CCL2) showed the highest baseline concentrations among the panel (median 108.3 pg/mL, range 58.1–201.9 pg/mL). Pro-inflammatory cytokines IL-8 and MIP-1β were also readily detectable at baseline (median 17.7 and 54.6 pg/mL, respectively). In contrast, Th1 cytokines (IFN-γ, IL-2) and Th2 cytokines (IL-4, IL-5, IL-13) were present at low or undetectable levels (Fig. S3 and S4).

#### Cytokine changes associated with HPV16 clearance

**Fig 2:**
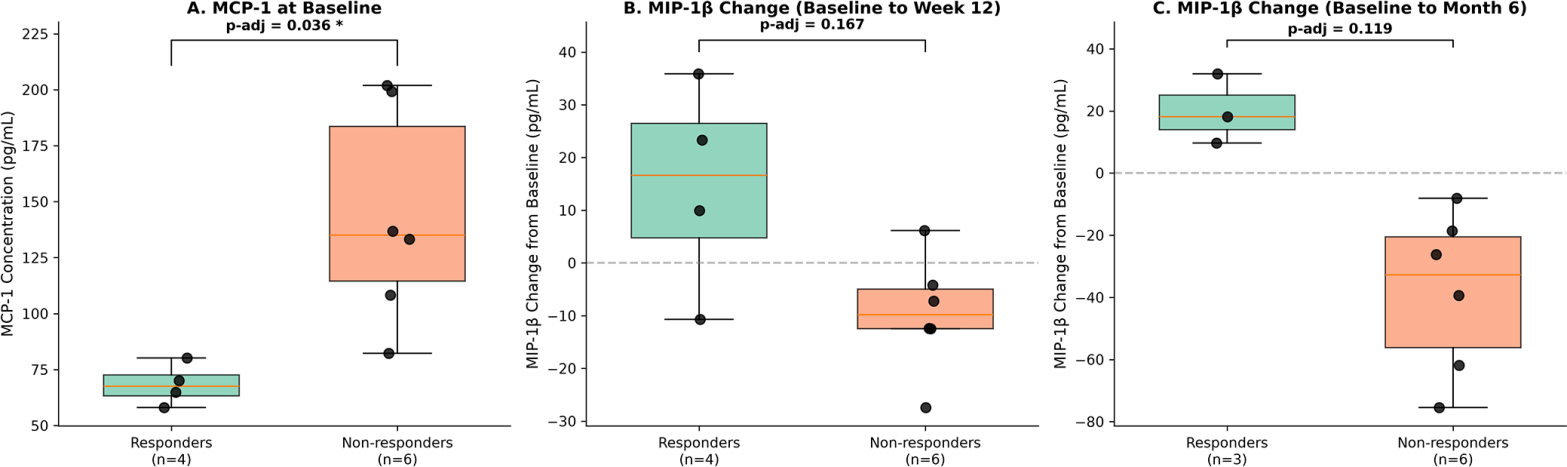
Serum cytokine profiles associated with HPV16 clearance. (**A**) Baseline MCP-1 concentrations in serum were significantly higher in non-responders compared with responders (*P*-adj = 0.036, Mann-Whitney U test with Benjamini-Hochberg correction). (**B**) Change in serum MIP-1β levels from baseline to week 12 in non-responders compared with responders compared by Mann-Whitney U test with Benjamini-Hochberg correction. (**C**) Change in serum MIP-1β levels from baseline to Month six compared by Mann-Whitney U test with Benjamini-Hochberg correction. Responders: *n* = 3–4; Non-responders: *n* = 6. One responder (1003-009) lacked baseline cytokine data; one responder (1002-003) was missing the Month six sample; one patient (1004-002) was lost to follow-up after week 12. Box plots show median with interquartile range.

To identify cytokine correlates of clinical response, we compared cytokine profiles between patients who cleared HPV16 (responders, *n* = 5) and those with persistent infection (non-responders, *n* = 6). One patient was lost to follow-up and was thus excluded from response comparisons. Comparisons of absolute concentrations were performed at each timepoint using the Mann-Whitney U test. Change-from-baseline comparisons were performed at Week 12 (all cytokines) and Month 6 (MIP-1β). At baseline, MCP-1 concentrations were significantly higher in non-responders compared with responders (*P* = 0.010, Mann-Whitney U test; Fig. 2). This difference suggests that elevated baseline systemic inflammation may be associated with impaired viral clearance. No other cytokines showed significant baseline differences between groups. MIP-1β (macrophage inflammatory protein-1 beta/CCL4) emerged as the most notable cytokine correlate of response when examining changes post vaccination. In responders serum MIP-1β levels increased from baseline to week 12 and month 6, while non-responders showed decreases (Fig. S4), but this did not reach statistical significance after correction for multiple comparison testing (Fig. 2). No significant differences were observed for IFN-γ, TNF-α, or other Th1/Th2 cytokines at any timepoint (Fig. S3 and S4), suggesting that the vaccine did not induce detectable systemic polarization of the T helper response. Several cytokines, including IL-1β, IL-4, and GM-CSF, were below the assay detection limit in most samples and could not be reliably analyzed.

### T-cell receptor repertoire dynamics

HPV16-specific T-cell responses are the expected driver of viral control. Prior ELISpot studies of the T-cell responses of patients to these vaccines administered alone three times suggested that they induced only weak CD8 T cell responses. As an alternative approach with greater sensitivity, we utilized TCRseq to monitor individual CD8 T cell clonotypes in peripheral blood mononuclear cells (PBMC).

#### Clonotype diversity and richness

Successful vaccination reshapes the TCR repertoire by clonal expansion of existing and *de novo* antigen-specific clonotypes rather than uniformly altering global T-cell diversity. Here, repertoire diversity was summarized with four standard indices, each capturing a different feature of the clonotype distribution: Shannon entropy (overall diversity, reflecting both the number of clonotypes and how evenly their frequencies are distributed), the inverse Simpson index (sensitivity to dominance by the most frequent clones), the Chao1 estimator (total richness, including rare clonotypes not directly observed), and the D50 index (the number of clonotypes accounting for 50% of all reads, a measure of repertoire concentration). We also report clonality (1 − normalized Shannon entropy), which ranges from 0 (an even, polyclonal repertoire) to 1 (monoclonal); higher clonality indicates dominance by fewer expanded clones. Full definitions are given in the supplemental methods.

TCRβ sequencing from peripheral blood yielded a median estimated richness (Chao1) of 24,846 clonotypes per sample (range: 12,193-36,112), reflecting the diverse T cell repertoire in circulation. Shannon diversity ranged from 6 to 2,788 across samples, with higher values indicating more evenly distributed repertoires. The Simpson index, which emphasizes dominant clones, ranged from 17 to 15,520. The D50 index, the minimum number of clonotypes comprising 50% of all reads, ranged from 427 to 6,583, with lower values indicating repertoires dominated by fewer expanded clones.

**Fig 3:**
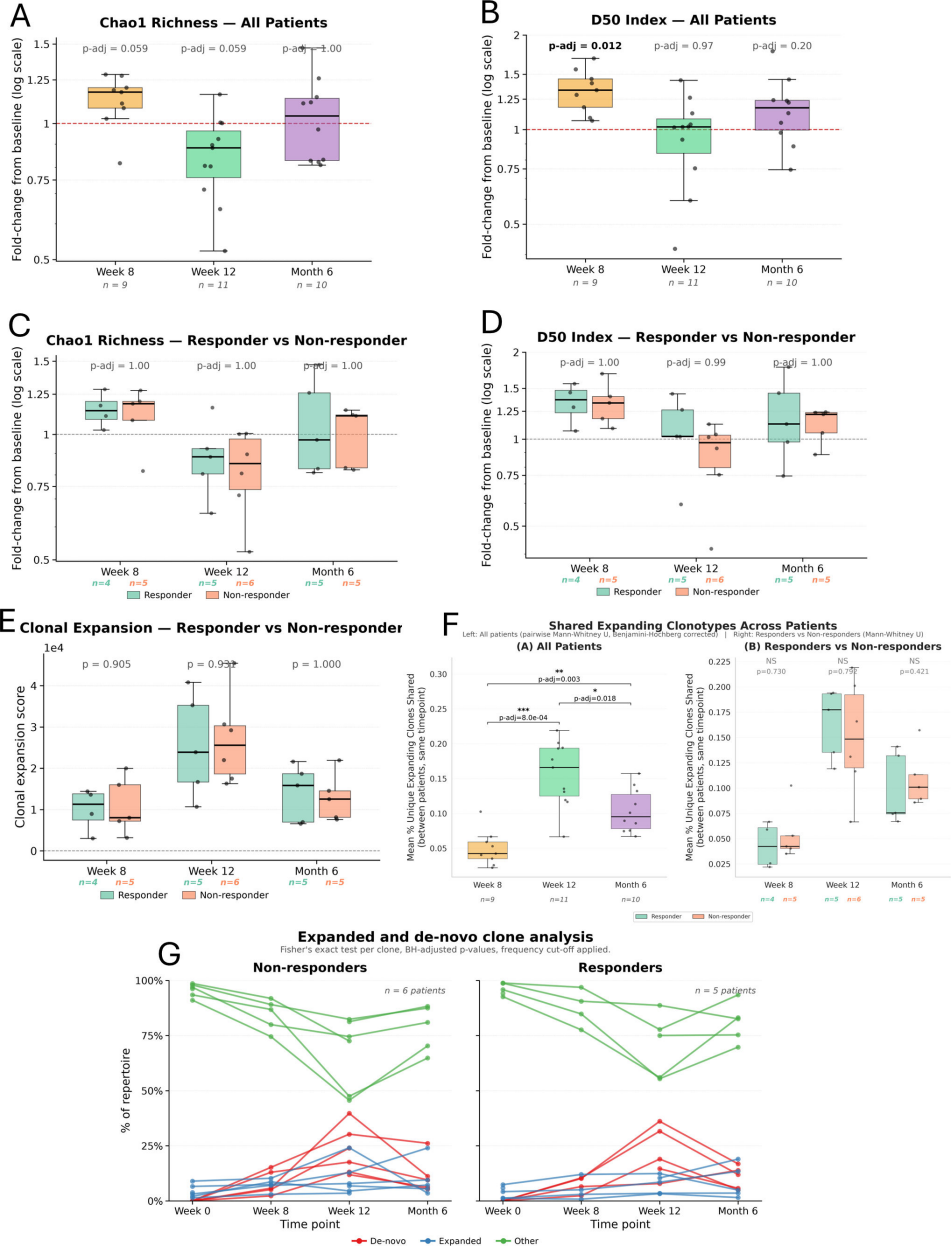
T cell receptor repertoire dynamics following PVX2 vaccination. (**A**–**D**) TCR repertoire diversity fold-change from baseline (Week 0) at post-vaccination timepoints (week 8, week 12, month 6): (**A**) Chao1 richness in all patients combined, (**B**) D50 index in all patients combined, (**C**) Chao1 richness comparing responders (green, #66C2A5) to non-responders (coral, #FC8D62), and (**D**) D50 index comparing responders to non-responders. For (A–B), dashed red line indicates no change from baseline (1×); p-values from one-sample Wilcoxon signed-rank test comparing fold-change to 1.0: Chao1 richness increased significantly at Week 8 (*P* = 0.039) and decreased at Week 12 (*P* = 0.032); D50 index increased significantly at Week 8 (*P* = 0.004). For (**C**–**D**), *P*-values from Mann-Whitney U test. No significant differences in fold-change between response groups at any timepoint. Shannon entropy and Simpson index were also analyzed but showed no significant changes and are reported in the text. (**E**) Cumulative expansion score (sum of log₂ fold-changes for all expanding clones) per patient at each post-vaccination timepoint. Responders (green) and non-responders (coral) showed similar expansion magnitudes with no significant differences between groups (Mann-Whitney U test *P*-values shown). Sample sizes for (**E**): week 8 (*n* = 4 responders, *n* = 5 non-responders; missing 1003-009, 1003-015), week 12 (*n* = 5 responders, *n* = 6 non-responders), Month 6 (*n* = 5 responders, *n* = 5 non-responders; missing 1003-008). (**F**) Between-patient sharing of expanding clonotypes across timepoints. All-patients sub-panel: percentage of each patient’s unique expanding clonotypes shared with other patients at the same timepoint for all 11 patients (excluding 1004-002); pairwise Mann-Whitney U with Bonferroni correction: sharing was significantly higher at week 12 vs Week 8 (*P* < 0.001) and month 6 vs week 8 (*P* = 0.005); Week 12 vs Month six trend (*P* = 0.055). Responder-vs-non-responder sub-panel: sharing comparison between responders (green, *n* = 4–5) and non-responders (coral, *n* = 5–6) at each timepoint; no significant differences (Mann-Whitney U p-values shown). Expanding clones defined by Fisher’s exact test (BH-adjusted *P* < 0.05) with positive fold-change from baseline. (**G**) Longitudinal dynamics of TCR repertoire composition classified by clonal expansion status (*de novo*, expanded pre-existing, other), shown separately for non-responders (left) and responders (right). The plots illustrate the shifting proportions of the circulating T-cell pool occupied by vaccine-induced (*De novo* / Expanded) versus stable clones over time. For all panels, box plots show median with interquartile range; individual data points are overlaid; sample sizes are indicated below each box. *n* = 11 patients (five responders, six non-responders; patient 1004-002 excluded due to loss to follow-up). Sample sizes for (A–D) vary by timepoint: week 8 (*n* = 9), week 12 (*n* = 11), month 6 (*n* = 10). Significance indicated by brackets where applicable: ***P < 0.001, **P < 0.01, *P < 0.05, ns = not significant.

To assess whether vaccination altered repertoire diversity, we calculated fold-change from baseline (week 0) for each diversity metric at post-vaccination timepoints (week 8, week 12, month 6). Of the four metrics examined (Shannon, Simpson, Chao1, D50), Chao1 richness and D50 index showed statistically significant changes from baseline, while Shannon entropy and Simpson index did not reach significance at any timepoint. Chao1 richness fold-change was significantly greater than 1.0 at week 8 (median 1.17, *P* = 0.039, Wilcoxon signed-rank test), indicating an early increase in estimated clonotype richness following DNA vaccination (Fig. 3A). By week 12, Chao1 richness significantly decreased below baseline (median 0.88, *P* = 0.032), suggesting a contraction phase following the initial expansion, before returning to baseline levels at Month 6 (median 1.04, *P* = 1.000). The D50 index showed significant increases at week 8 (median 1.34, *P* = 0.004), indicating that more clonotypes were required to account for 50% of reads, consistent with a more evenly distributed repertoire early after vaccination (Fig. 3B). D50 returned to baseline levels by week 12 (median 1.02, *P* = 0.966) and month 6 (median 1.17, *P* = 0.131). Shannon entropy and Simpson index showed modest non-significant increases across timepoints (all *P* > 0.10), consistent with their lower sensitivity to changes in rare clonotypes. When comparing fold-change between responders and non-responders, no significant differences were observed for either Chao1 or D50 at any timepoint (Mann-Whitney U test, all *P* > 0.30; Fig. 3C and D), indicating that vaccination-induced diversity changes were similar regardless of clinical outcome. Cross-sectional comparison of raw diversity values at individual timepoints also showed no significant differences between groups (all adjusted *P* > 0.10, Mann-Whitney U test with Benjamini-Hochberg correction). Patient 1003-001, a responder, consistently showed the highest diversity (Shannon entropy 2,720 at baseline, 1,608 at Week 12), while Patient 1003-009, also a responder, had notably low diversity (Shannon entropy 6.0–7.5 across timepoints), suggesting a highly oligoclonal repertoire. One notable exception to within-patient stability was Patient 1004-001 (non-responder), who exhibited a dramatic transient oligoclonal expansion at week 12 (Shannon diversity 9.6 vs 30–120 at other timepoints; clonality 0.19 vs 0.08–0.09) that resolved by month 6, visible as an outlier in the PCA ordination (Fig. S5).

We also calculated clonality (1 − normalized Shannon entropy), which provides a complementary measure of repertoire focus. Higher clonality indicates a more oligoclonal repertoire dominated by fewer expanded clones. When analyzed as fold-change from baseline, identical to the approach used for other diversity metrics (Fig. 3A through D), clonality showed a significant decrease at Week 8 (median fold-change 0.91, *P* = 0.008, Wilcoxon signed-rank test vs 1.0), indicating that repertoires became more polyclonal early after vaccination, consistent with the diversity increases observed for Chao1 and D50 at the same timepoint (Fig. 3A and B). By week 12, clonality had returned toward baseline (median 0.94, *P* = 0.320), and at month 6 it remained modestly below baseline (median 0.89, *P* = 0.160), though neither change was statistically significant (Fig. S6A). Comparison of clonality fold-change between responders and non-responders revealed no significant differences at any timepoint (Mann-Whitney U test, all *P* > 0.50; Fig. S6B), consistent with the other diversity metrics.

#### Repertoire composition differs between response groups

To assess whether overall repertoire composition differed between responders and non-responders, we performed permutational multivariate analysis of variance (PERMANOVA) on PCA-based distance matrices (Fig. S5 and S7). We tested multiple clinical and demographic factors to comprehensively characterize sources of repertoire variation. As expected, individual patient identity explained the largest proportion of variance (R² = 0.98, *P* = 0.001), reflecting the highly personalized nature of TCR repertoires shaped by each individual’s HLA genotype, infection history, and stochastic V(D)J recombination. After accounting for this expected inter-individual variation, treatment response emerged as the strongest biologically meaningful factor, explaining 54.1% of variance (R² = 0.54, *P* = 0.005). In contrast, vaccination timepoint did not significantly explain repertoire variation (R² = 0.75, *P* = 0.998), indicating that repertoire composition remained relatively stable over the 6-month study period despite vaccination-induced clonal expansions. Age group (R² = 0.49, *P* = 0.43) and ethnicity (R² = 0.48, *P* = 0.78) were not significant predictors of repertoire composition. Race showed a significant effect (R² = 0.35, *P* = 0.002), likely driven by the single Asian patient (1004-001) who exhibited distinctive repertoire characteristics, including the transient oligoclonal expansion at week 12. Because the six factors were each tested independently and not corrected across factors, these *P*-values are nominal; the treatment-response effect nonetheless remains significant under Benjamini-Hochberg correction across the factors tested (q ≈ 0.01). These results indicate that clinical response status is associated with overall repertoire structure, supporting the hypothesis that pre-existing or vaccination-induced repertoire features differ between responders and non-responders.

#### Substantial clonal expansion after vaccination

To identify vaccine-induced T-cell responses, we tracked clonotype dynamics from baseline through post-vaccination timepoints. Expanding clonotypes were identified using a one-sided Fisher’s exact test on each clonotype’s 2 × 2 contingency table of read counts (Benjamini-Hochberg adjusted *P* < 0.05; see Methods), adapted from Zhang et al. (40), and further classified as *de novo* (absent at baseline, emerging post-vaccination) or expanded pre-existing (detectable at baseline with significant post-vaccination expansion). Substantial clonal expansion was observed in all patients, with 6,319 expanding clonotypes identified at week 8, 19,137 at week 12 (peak expansion), and 8,983 at month 6 across all patients (including patient 1004-002, who was subsequently lost to follow-up). The majority of expanding clones (~89%) were *de novo* clonotypes (absent at baseline but appearing post-vaccination), while ~11% were pre-existing clones that expanded following vaccination. Among the 11 patients with treatment response classification, week 12 expansion comprised 16,734 *de novo* and 1,457 pre-existing expanded clonotypes (92% *de novo*). Both responders and non-responders showed similar numbers of expanding clones at each timepoint (Fig. 3E). Comparison of cumulative expansion scores (sum of log₂ fold-changes for all expanding clones per patient) revealed no significant differences between responders and non-responders at week 8 (*P* = 0.91), week 12 (*P* = 0.93), or month 6 (*P* = 1.00, Mann-Whitney U test). This indicates that vaccination induced substantial T-cell activation regardless of clinical outcome, and the distinguishing features between groups emerged in the identity and specificity of expanding clones rather than the overall magnitude of expansion. Analysis of repertoire composition revealed that *de novo* clones comprised a substantial fraction of post-vaccination repertoires in both response groups, with no significant difference in the proportion of *de novo* versus expanded pre-existing clones between responders and non-responders at any timepoint (Fig. S8). Longitudinal analysis of these repertoire composition trajectories (Fig. 3G) further illustrated the dynamic balance between *de novo* and pre-existing expanded clones throughout the vaccination period.

#### Between-patient convergence of expanding clones

To assess the extent of convergent T-cell responses across the cohort, we examined expanding clones shared between multiple patients, i.e., “public” clonotypes, which arise independently in more than one individual, in contrast to “private” clonotypes found in only one person. We identified 149 unique CDR3 sequences that expanded (Fisher’s exact test, BH-adjusted *P* < 0.05) in two or more patients at the same post-vaccination timepoint. Pairwise comparisons revealed that between-patient sharing of expanding clones differed significantly across timepoints (Fig. 3F, all-patients sub-panel): sharing was significantly higher at Week 12 than Week 8 (*P* < 0.001) and Month six than week 8 (*P* = 0.005), while the week 12 vs month 6 comparison showed a trend (*P* = 0.055; all Mann-Whitney U with Bonferroni correction). Sharing peaked at week 12 (105 shared clones) compared with week 8 (11 clones) and month 6 (36 clones). The timing of peak sharing coincided with peak clonal expansion, suggesting that the vaccine-induced T-cell response converges on a common set of clonotypes at the height of immune activation. Notably, the degree of between-patient sharing did not differ significantly between responders and non-responders at any timepoint (Mann-Whitney U, all *P* > 0.7; Fig. 3F, responder-vs-non-responder sub-panel), indicating that public clone sharing is a general feature of vaccination rather than a correlate of HPV16 clearance.

Among the 105 shared expanding clones at week 12, the majority (68; 64.8%) were shared between both response groups (R + NR), while 17 (16.2%) were responder-only and 20 (19.0%) were non-responder-only. Cross-referencing these shared expanding clones with MANAFEST peptide-stimulation data revealed that only 2 of 149 unique shared CDR3 sequences (1.3%) were identifiable as antigen-reactive in the MANAFEST assay, both reactive to HPV16 E6 peptides at week 12 (CASSLGGNQPQHF, shared by four patients; CASSLEGNQPQHF, shared by two patients). This low overlap is consistent with the overall rarity of MANAFEST-identifiable clones in peripheral blood and suggests that the majority of shared expanding clones may represent bystander activation (i.e., T-cell expansion driven by the inflammatory milieu of vaccination rather than directly by an HPV peptide) or responses to epitopes not captured by the MANAFEST peptide panel.

We additionally identified 22 CDR3 sequences that expanded in two or more responders but not in any non-responder across any timepoint. These responder-specific shared clones were each shared by 2–3 responders, with expansion magnitudes ranging from modest (log₂ FC 1.3) to very large (log₂ FC 17.8, corresponding to >200,000-fold). Their restriction to the responder group makes them candidates for HPV16-driven T-cell responses, though as with the broader set of shared expanding clones, the majority could not be confirmed as antigen-specific by the MANAFEST assay.

#### V and J gene usage patterns

Analysis of TRBV gene usage revealed subtle but consistent differences between responders and non-responders (Fig. S9 and S10). The most abundant V genes across all samples were TRBV20-1 (11.1% of repertoire), TRBV19 (6.6%), TRBV28 (5.7%), TRBV7-9 (5.2%), and TRBV27 (4.5%). Temporal patterns of V gene usage were relatively stable across timepoints (Fig. S9), with only TRBV18 (*P* = 0.048) and TRBV15 (*P* = 0.007) showing significant changes over time (Kruskal-Wallis test; Fig. S10A). Several V genes showed differential usage between response groups (Fig. S10B). TRBV6-4 was enriched in responders (1.52% vs 0.97%, log₂ FC = +0.64), as was TRBV28 (6.61% vs 5.00%, log₂ FC = +0.40). Conversely, TRBV11-3 was underrepresented in responders (0.95% vs 1.67%, log₂ FC = −0.81), representing the largest differential between groups. TRBV7-2 was also lower in responders (3.78% vs 4.96%, log₂ FC = −0.39). Examination of V gene usage among the shared responding clones revealed frequent use of TRBV27 (18 of 49 gene assignments), TRBV20-1 (9), TRBV7-9 (4), TRBV7-2 (3), and TRBV6 family genes (TRBV6-1, TRBV6-5, TRBV6-6; two each). These represent commonly used V genes in the human TCRβ repertoire, consistent with vaccine-induced expansion of pre-existing memory or naive T-cell populations rather than *de novo* generation of public TCR sequences. Among TRBJ genes, TRBJ2-7 was the most frequently used (15.3%), followed by TRBJ1-1 (13.8%) and TRBJ1-2 (12.6%) (Fig. S11). TRBJ1-4 showed a significant increase over time (*P* = 0.016, Kruskal-Wallis), rising from 1.8% at baseline to 2.9% at month 6 (Fig. S12).

#### CDR3 length distribution

CDR3 length distributions were highly consistent across samples, with mean lengths ranging from 14.3 to 14.8 amino acids and modal lengths of 14–15 amino acids. CDR3 length distributions did not differ significantly between responders and non-responders at any timepoint (all *P* > 0.40, Mann-Whitney U test). This indicates that while individual clones within the repertoire showed differential expansion, the overall structural properties of the TCR repertoire as measured by CDR3 spectratype remained stable.

#### CDR3 sequence motifs associated with response

Analysis of 3-mer amino acid motifs within CDR3 sequences revealed sequence patterns differentially enriched between responders and non-responders. Among the most statistically significant differentially enriched motifs (Mann-Whitney U test), responders showed higher frequencies of “TSD” (*P* = 8.3 × 10⁻⁷), “EVN” (*P* = 3.9 × 10⁻⁶), “GHT” (*P* = 6.5 × 10⁻⁶), “ELY” (*P* = 1.1 × 10⁻⁵), and “HYY” (log₂ FC = +2.4, *P* = 7.2 × 10⁻⁵). Conversely, non-responders showed enrichment of motifs with larger fold-changes, including “CQY” (log₂ FC = −20.0, *P* = 0.008), “YHW” (log₂ FC = −19.7, *P* = 0.008), and “EPF” (log₂ FC = −4.6, *P* = 0.004). While individual 3-mer associations require validation with larger cohorts, these findings suggest that CDR3 sequence features may predict vaccine response and provide molecular signatures for further investigation.

### Antigen specificity validation by MANAFEST

The HPV-specificity was assessed by MANAFEST, in which pools of CD8 T cells are stimulated with overlapping pools of HPV E6, E7, or L2 peptides, and their expansion detected by TCRseq. Specifically, we analyzed samples from 12 patients (including technical replicates for patient 1003-008) across 13 peptide conditions to identify antigen-specific clonotypes. Using negative binomial regression with stringent statistical criteria (FDR < 0.05, OR ≥ 1), we identified 19,689 unique putative HPV-specific clonotypes across all patients based upon significant enrichment in at least one HPV peptide condition while not meeting enrichment criteria in a negative control (HIV peptides to exclude non-specific responses). These putative HPV-specific clones were distributed across all three viral antigens: 5,368 E6-reactive clones (27.3%), 7,685 E7-reactive clones (39.0%), and 6,636 L2-reactive clones (33.7%) (Table 1). The median odds ratio among HPV-specific clones was 70.4 (range: 1.2–10,530.5), indicating robust enrichment relative to unstimulated controls.

**TABLE 1:** HPV-specific clones by patient and peptide from MANAFEST analysis^*a*^

Study ID	Treatment response	E6 clones	E7 clones	L2 clones	Total HPV clones
1002-002	Non-responder	432	337	424	1,193
1002-003	Responder	505	497	494	1,496
1003-007	Responder	145	193	120	458
1003-008	Non-responder	560	426	832	1,818
1003-008r	Non-responder	471	598	363	1,432
1003-009	Responder	97	86	126	309
1003-001	Responder	648	434	258	1,340
1003-006	Non-responder	32	345	274	651
1003-002	Non-responder	1,348	3,130	2,179	6,657
1004-003	Responder	334	604	713	1,651
1004-001	Non-responder	388	492	472	1,352
1004-002	Excluded	160	245	158	563
1003-015	Non-responder	248	298	223	769

^*a*^ Distribution of statistically significant HPV-specific clonotypes (negative binomial regression, FDR < 0.05, OR ≥ 1) identified by peptide-stimulation TCRseq across 13 patients. Columns show: Patient ID, treatment response status (responder/non-responder), number of E6-reactive clones, E7-reactive clones, L2-reactive clones, and total HPV-specific clones. Totals: 5,368 E6-reactive, 7,685 E7-reactive, 6,636 L2-reactive, 19,689 total HPV-specific clones. Technical replicates from 1003 to 018 are shown separately (1003-008r).

#### Patient-level distribution of HPV-specific clones

The number of putative HPV-specific clones varied substantially between patients (Table 1). Clone counts ranged from 309 (patient 1003-009, responder) to 6,657 (patient 1003-002, non-responder). Among responders (*n* = 5), a total of 5,254 HPV-specific clones were identified (mean 1,050.8 per patient; range 309-1,651). Among non-responders (*n* = 6), 12,440 HPV-specific clones were identified (mean 2,073 per patient; range 651-6,657), with the higher mean driven primarily by a single outlier (patient 1003-002). Clone counts did not differ significantly between groups (*P* = 0.54, Mann-Whitney U test), consistent with the overall finding that the identity rather than quantity of antigen-specific responses may distinguish clinical outcomes.

The technical replicates (patient 1003-008) demonstrated assay reproducibility: the first replicate yielded 1,818 HPV-specific clones, and the second 1,432 clones. Despite similar total counts, only 14 CDR3 sequences were shared between replicates (0.4% Jaccard overlap; Table 1), indicating that each replicate captured a largely distinct subset of the HPV-reactive repertoire. This low overlap is consistent with small number of cells/well used for *in vitro* peptide stimulation and the relatively low frequency of HPV-specific T cells in circulation.

#### Cross-reference of genomic TCR repertoire with MANAFEST antigen specificity

To assess whether clones detected in peripheral blood were specific for HPV antigens, we cross-referenced all clonotypes from longitudinal bulk TCRseq with the MANAFEST antigen-specific clone database using matching CDR3 amino acid sequences within the same patient. To account for inter-patient differences in sequencing depth, all comparisons used fraction-based metrics (expressing antigen-specific reads as a percentage of each patient’s total repertoire) rather than absolute counts.

Across all patients and timepoints, a small but reproducible fraction of clones were confirmed as antigen-specific by MANAFEST. Of 1,330,170 total genomic clonotypes across 12 patients and four timepoints, 3,936 (0.30%) were identified as antigen-specific by CDR3 match to the MANAFEST database within the same patient, comprising 840 E6-specific, 783 E7-specific, 783 L2-specific, and 1,536 CEF-specific clones. The antigen-specific fraction of total reads peaked at Week 12 (0.59%), coinciding with the peak of overall clonal expansion, and remained detectable at Month 6 (0.57%), suggesting persistence of antigen-driven T cells in circulation. Examining the overlap from the MANAFEST perspective, 3,991 of the 19,689 HPV-specific clonotypes (20.3%) were detected in at least one genomic TCRseq sample from any patient, and 1,404 (7.1%) were found in the same patient’s peripheral blood (Table 2), providing direct evidence that MANAFEST-identified antigen-specific T cells circulate *in vivo*.

**TABLE 2:** Cross-reference of MANAFEST HPV**-**specific clones with peripheral blood TCRseq^*a*^

Study ID	Response	MANAFEST HPV clonotypes	Found any genomic	Pct any genomic	Found same patient	Pct same patient	E6 same patient	E7 same patient	L2 same patient
1002-003	Responder	1,496	281	18.8	74	4.9	24	24	26
1003-007	Responder	458	80	17.5	22	4.8	7	11	4
1003-009	Responder	309	87	28.2	46	14.9	15	14	17
1003-001	Responder	1,340	371	27.7	245	18.3	147	45	53
1004-003	Responder	1,651	368	22.3	193	11.7	51	64	78
1002-002	Non-responder	1,193	229	19.2	96	8	36	27	33
1003-008	Non-responder	1,818	345	19	110	6.1	28	25	57
1003-006	Non-responder	651	164	25.2	81	12.4	21	33	27
1003-002	Non-responder	6,657	1,285	19.3	404	6.1	70	200	134
1004-001	Non-responder	1,352	256	18.9	56	4.1	23	19	14
1003-015	Non-responder	769	140	18.2	46	6	9	19	18
1004-002	Excluded	563	97	17.2	31	5.5	7	15	9

^*a*^ Validation of MANAFEST-identified HPV-specific clones in longitudinal bulk TCRseq data from PBMCs (12 patients; technical replicate of 1003-008 excluded). Columns show: Patient ID, treatment response, total HPV-specific clonotypes from MANAFEST, number found in any genomic sample (any patient), percentage found in any genomic sample, number found in the same patient’s genomic samples, percentage same-patient overlap, and breakdown of same-patient matches by antigen (E6, E7, L2). Patient 1004-002 included as excluded (lost to follow-up). Same-patient overlap ranged from 4.1% (1004-001) to 18.3% (1003-001). Overall, 3,991 of 19,689 HPV-specific clonotypes (20.3%) were detected in at least one genomic sample, and 1,404 (7.1%) in the same patient’s peripheral blood.

#### Responder versus non-responder comparison

Comparison of the antigen-specific read fraction between responders and non-responders revealed that responders exhibited a higher proportion of antigen-specific reads among their total repertoire at post-vaccination timepoints, although comparisons at individual timepoints did not reach statistical significance with the available sample size (Mann-Whitney U test). This pattern was consistent across reads-based metrics: the percentage of total reads that were antigen-specific trended higher in responders. Breaking down the antigen-specific response by individual HPV antigen and the CEF-positive control revealed antigen-specific patterns (Fig. S13A through D). E6-specific reads showed a trend toward higher fractions in responders at Week 12 (Fig. S13A), consistent with the anti-E6 antibody response that also distinguished responders (Fig. 1E). E7-specific responses were detectable in both groups (Fig. S13B), while L2-specific reads showed variable patterns across timepoints (Fig. S13C). CEF-reactive reads, representing pre-existing memory responses to common viral antigens, showed comparable fractions between groups (Fig. S13D), as expected for a non-vaccine-specific control.

#### *In silico* validation of MANAFEST specificity

To validate the biological specificity of the MANAFEST assay, we assessed whether CEF-reactive clones (enriched following stimulation with CMV/EBV/influenza peptides) were independently identifiable as CMV/EBV/influenza-reactive in the VDJdb public TCR–epitope database. MANAFEST-identified CEF clones found in the genomic repertoire were significantly enriched for exact CDR3β sequence matches to CMV/EBV/influenza entries in VDJdb compared with non-CEF genomic clones (9.0% vs 0.5%; OR = 18.7, *P* = 3.58 × 10⁻⁶⁹, Fisher’s exact test; Fig. S14). This enrichment supports the validity of the MANAFEST assay for identifying antigen-specific T cells, providing confidence in the HPV-specific clone assignments used throughout this analysis.

## DISCUSSION

Humoral and T-cell responses to PVX2 vaccination were evident in HPV16+ <CIN2 patients. The E7-specific antibody response to the DNA vaccination was weak, which likely reflects its delivery to the muscle without electroporation, and the fusion of E7 to HSP70, a molecular adjuvant that promotes CD8 + T cells but not antibody responses in mouse models. Antibody responses to L2 and E6 were boosted by 1 month after a single dose of TA-CIN and E7 one month after the second DNA vaccination. Again, these were weak and waned, likely reflecting the administration of TA-CIN without an adjuvant and DNA without electroporation. Only the E6 antibody response at week 12 was associated with viral clearance, but this did not survive multiple-testing correction. Control of an intracellular pathogen, such as HPV16 is unlikely to be mediated by E6 antibodies, but potentially they serve as a biomarker for an E6-specific CD4 T cell response.

Examination of the TCR repertoire dynamics revealed significant temporal diversity changes that follow a biologically coherent pattern. Chao1 richness increased significantly at week 8 (*P* = 0.039), then decreased below baseline at week 12 (*P* = 0.032), before returning to baseline at month 6. D50 increased at week 8 (*P* = 0.004). Clonality decreased at week 8 (*P* = 0.008). This arc of expansion, contraction, and recovery is consistent with initial polyclonal activation of diverse clones after DNA vaccination, then selection/contraction as the immune response focuses on relevant clones after the TA-CIN protein boost (week 12), followed by homeostatic recovery (month 6). However, the TCR repertoire dynamics in both the group of patients that clear HPV16 and those that do not follow the same trajectory.

Analysis of TCR repertoire by PERMANOVA suggests clinical response status is associated with overall repertoire structure, supporting the hypothesis that pre-existing and/or vaccination-induced repertoire features differ between responders and non-responders (41, 42). Standard diversity indices (Shannon, Simpson, Chao1, D50) measure how many and how evenly distributed clones are. These are “quantity” metrics. PERMANOVA on PCA-transformed data captures which specific clones are present and at what frequencies: a “quality” or “identity” metric. The fact that composition (R² = 0.54, *P* = 0.005) distinguishes groups, while diversity does not, indicates that responders and non-responders have repertoires of similar complexity but fundamentally different clonal content. As expected, patient identity explains 98% of repertoire variance reflecting the highly personalized nature of TCR repertoires. Despite this dominant patient effect (R² = 0.98, *P* = 0.001), treatment response still emerges as significant (*P* = 0.005) within this overwhelming individual variation. This indicates the response-associated signal is detectable even within this dominant individual variation (43).

All patients showed substantial expansion (~19,000 clones at week 12, mean log₂ FC ~15). The fact that cumulative expansion scores were identical between groups (*P* = 0.91–1.00 at all timepoints) suggests that PVX2 vaccination activates T cells robustly regardless of whether HPV16 cleared or not. While this could imply that clearance is spontaneous or not CD8 T-cell-mediated, an alternative possibility is that the distinguishing feature lies in the specificity of expanding CD8 T-cell clones, i.e., whether they target HPV antigens. PVX2 vaccination induces substantial clonal expansion comprising ~89% *de novo* clones (i.e., not detected at baseline) and ~11% expanded pre-existing. The dominance of *de novo* clonotypes suggests these patients had a weak prior T-cell response, likely a reflection of the very localized infection by this stealthy virus. In addition, it is possible that the HPV16-specific T cells accumulated at the site of infection rather than circulating in peripheral blood, as seen for other systemic viral infections like CMV, EBV, or influenza. Presumably, PVX2 vaccine generates these *de novo* T cells from the naive precursor pool (true *de novo*), but it is possible that these clones were present at baseline but below our sequencing detection limit (false *de novo*) and/or resident in the genital mucosa.

Public clonotypes that expand in multiple patients provide the strongest evidence for antigen-driven (rather than stochastic) expansion (43–45). The 149 shared expanding CDR3s, and particularly the peak at week 12 (105 clones), suggest that the vaccine is driving convergent T-cell responses across the cohort. However, sharing did not differ between R vs NR, implying the convergent clones are not response-specific. They may represent (i) common viral epitope responses shared by patients with similar HLA alleles, (ii) “innate-like” T cells, such as mucosal-associated invariant T (MAIT) cells and natural killer T (NKT) cells, which express semi-invariant TCRs shared across individuals, or (iii) bystander expansions driven by the inflammatory milieu of vaccination (46, 47). The 22 CDR3 sequences expanded in two to three responders but zero non-responders suggest their importance in viral clearance, but their specificity needs to be verified experimentally.

The analysis of serum cytokine levels revealed that an elevated level of monocyte chemoattractant protein-1 (MCP-1, also known as CCL2) level at baseline is associated with viral persistence. Indeed, elevated serum levels of MCP-1 are evident in cervical and other cancer patients, and in both, correlated with poor clinical outcomes (48). MCP-1 has been linked to chronic inflammation and an immunosuppressive tumor microenvironment, and to resistance to immunotherapies, such as immune checkpoint inhibitors (e.g., anti-PD-1), by recruiting cells that block antitumor immunity. MCP-1 is a potent chemoattractant for monocytes, which differentiate into tumor-associated macrophages (TAMs), specifically the pro-tumor M2 phenotype. MCP-1 is a key driver in the recruitment of myeloid-derived suppressor cells (MDSCs) that inhibit the activation and cytotoxicity of CD8+ effector T cells. MCP-1 also recruits regulatory T cells, which further suppress the immune system’s ability to attack tumor cells (48, 49). Whether any of these cancer-associated mechanisms operate in low-grade HPV16 infection is unknown; however, the observed association between higher baseline MCP-1 and HPV16 persistence suggests this possibility warrants further exploration.

Serum levels of MIP-1β trend higher post-vaccination in responders at week 12 and month 6, while in non-responders they decline (Fig. S4). This pattern could reflect the induction of a systemic cell-mediated immune response and its recruitment to the site of infection to control the virus in responders, and its suppression in non-responders. In this model, high MCP-1 levels in non-responders at baseline recruits immunosuppressive myeloid cells (M2 TAMs, MDSCs, Tregs), preventing an effective response, while MIP-1β increasing in responders post-vaccination recruits effector cells (CD8+ T cells, NK cells, DCs) to clear the virus (48, 49). In addition, since HPV infections are not systemic but highly localized within the cervical epithelium, the observed cytokine effects may be better measured through sampling locally (50, 51).

Interpreting HPV clearance after PVX2 immunotherapy requires comparison with a placebo. In the absence of this, some context may be gained by examination of the natural history of HPV16 in women with low-grade cytologic abnormality. At the month 6 primary endpoint, 5 of 11 evaluable participants (45%) had cleared HPV16, and all five remained HPV16-negative at month 12. In a natural history study of older women (mean 38.5 years, close to mean 42.5 years of the PVX2 participants) with low-grade cytologic abnormality, spontaneous HPV16 clearance occurred in 9% (95% CI 5–17) at 6 months, and only 19% (95% CI 12–29) at 18 months, markedly lower than the 45% clearance observed in the small PVX2 study. Nonetheless, because this was an uncontrolled, single-arm cohort of only 11 evaluable women, clearance cannot be attributed to the intervention. These observations should therefore be regarded as a hypothesis-generating signal that warrants evaluation in a randomized, controlled trial.

Limitations of this study include its small sample size that limits statistical power and generalizability, and the lack of a placebo control group. Measurement of serum cytokines are systemic proxies of the functional cervical tissue-level cytokine profiles, and they may differ dramatically. Likewise, bulk TCRβ sequencing from PBMCs misses the tissue-resident repertoire and does not provide TCRα pairing information. The MANAFEST uses small numbers of PBMCs which, for low-frequency CD8 T cells, results in significant under-sampling.

Future directions include electroporation-enhanced delivery of the DNA vaccine, targeting E6 and L2 in addition to E7 in the DNA to improve priming of the HPV-specific T-cell responses, development of an adjuvanted TA-CIN formulation, and consideration of modulation of the MCP-1/CCR2 axis to improve efficacy.

## Data Availability

De-identified individual participant data and the study protocol may be made available to qualified researchers for non-commercial, academic research. Due to commercial sensitivity and intellectual property restrictions, data requests will be reviewed by a joint steering committee. Requests must be sent to PapiVax Biotech Inc. (https://papivax.com/contact-us/) with a formal research proposal. Approved requestors will be required to sign a strict Data Use Agreement (DUA) that protects participant and commercial confidentiality, proprietary rights. Numerical data for all biological experiments associated with the individual figures are available from the corresponding author upon request.

## SUPPLEMENTAL MATERIAL

Supplemental material jvi.00643-26-s0001.pdf Fig. S1 to S14 and supplemental methods.

ASM does not own the copyrights to Supplemental Material that may be linked to, or accessed through, an article. The authors have granted ASM a non-exclusive, world-wide license to publish the Supplemental Material files. Please contact the corresponding author directly for reuse.
