# Supplementary material for: Exploration of immune correlates of viral clearance after PVX2 immunotherapy in patients with human papillomavirus type 16 infection and cervical intraepithelial neoplasia grade ≤1
Source: J Virol. 2026 Jul 27;100(8):e00643-26. doi: 10.1128/jvi.00643-26 (PMC13483411; doi:10.1128/jvi.00643-26)
Supplement: Supplementary file 1 [file jvi.00643-26-s0001.pdf]

Table of Contents

Supplementary Figures ... 1
Supplementary Methods ... 14
HPV16 E6, E7 and L2 Antibody Response Assessment by ELISA ... 14
Serum Cytokine Profiling by Luminex Multiplex Assay ... 15
T Cell Receptor Sequencing from Peripheral Blood ... 16
Repertoire Analysis ... 16
Clonal Expansion Analysis ... 17
Peptide-Stimulation TCR Sequencing (MANAFEST Assay) ... 18
References ... 20

Supplementary Figures

Figure S1

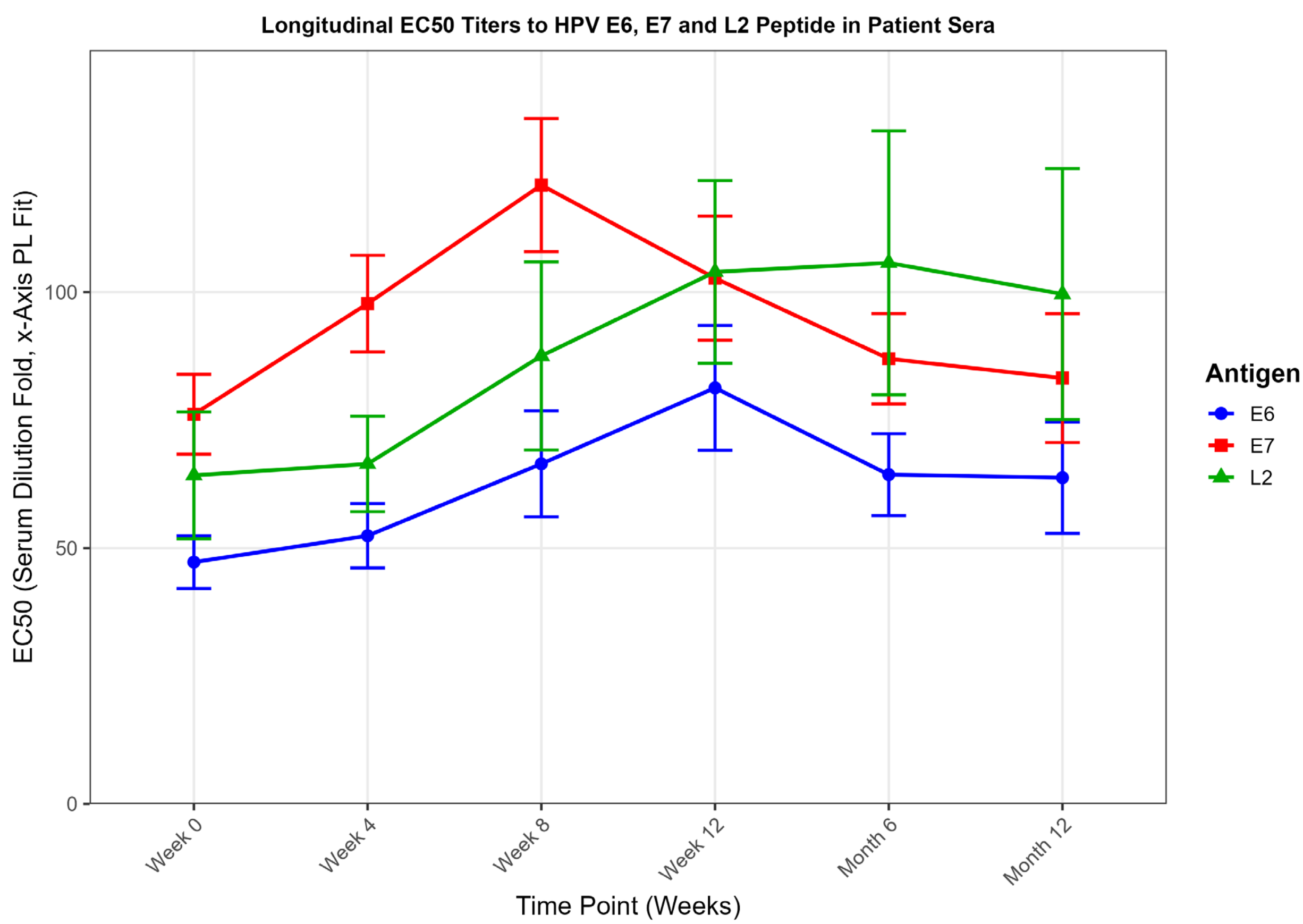

Figure S1. Longitudinal absolute EC_{50} values for HPV16 antibody responses. Absolute serum IgG antibody titers (EC_{50}) against HPV16 E6, E7, and L2 proteins measured by ELISA at baseline (Week 0), during vaccination (Weeks 4, 8), post-vaccination (Week 12), and follow-up (Months 6, 12). Lines

represent median EC_{50} values with interquartile range. Higher EC_{50} values indicate stronger antibody responses. n=11 patients.

Figure S2

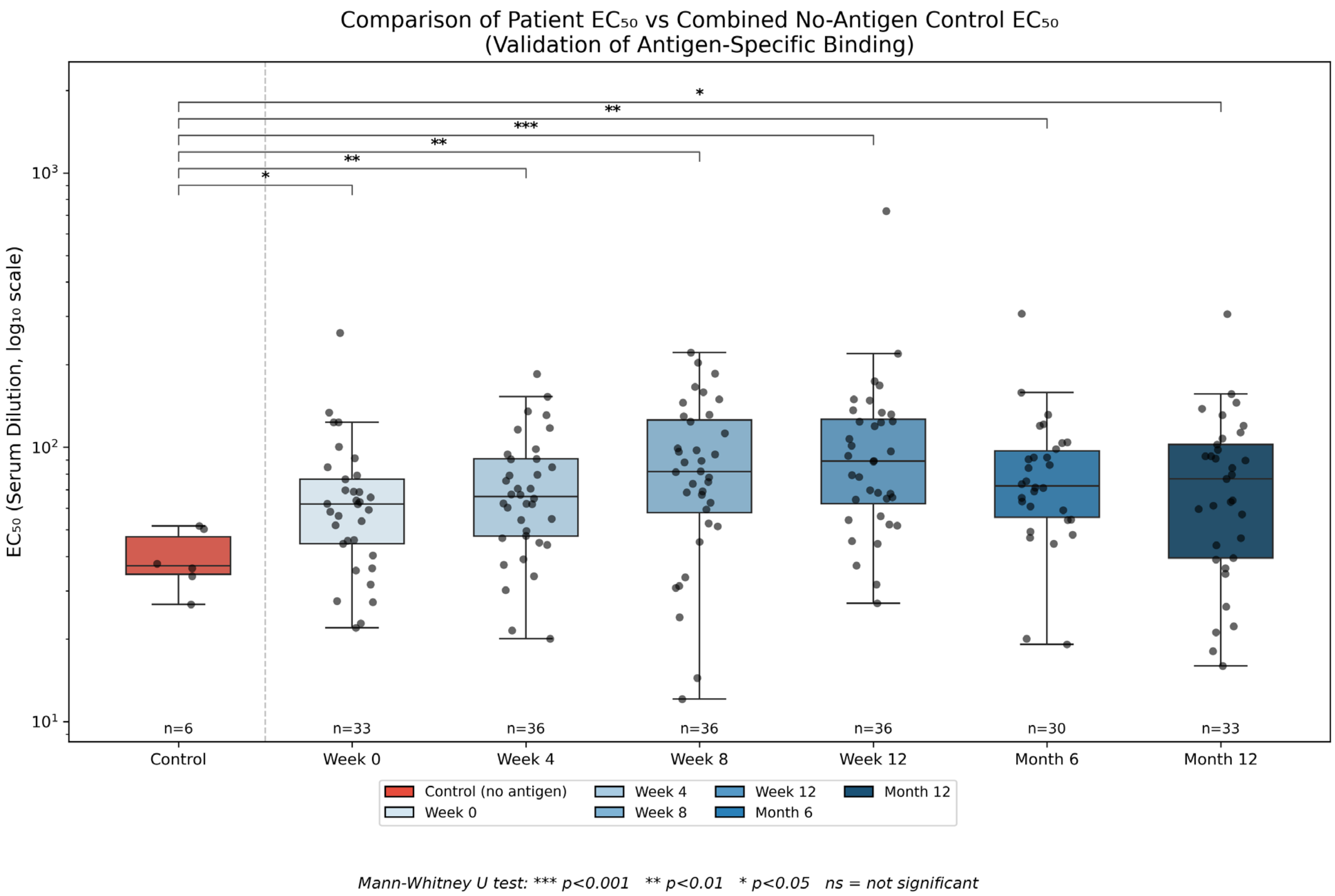

Figure S2. Validation of antigen-specific antibody detection. Comparison of patient EC_{50} values against no-antigen controls at all study timepoints. Control samples consisted of week 0 serum of 1004-002 tested against buffer-coated wells (no HPV16 antigen), representing non-specific binding background. Red box shows combined controls (n=6); blue boxes show patient samples at each timepoint with color gradient indicating temporal progression (light to dark: Week 0, Week 4, Week 8, Week 12, Month 6, Month 12). Patient EC_{50} values were significantly higher than controls at all timepoints (Mann-Whitney U test), including baseline (Week 0: ratio 1.73×, $p = 0.025$), confirming that detected antibody responses represent true antigen-specific binding rather than assay background. The patient-to-control ratio increased following vaccination, peaking at Week 12 (ratio 2.83×, $p < 0.001$). Boxes show median with interquartile range; individual data points overlaid. Y-axis shown on log scale. ***$p < 0.001$, **$p < 0.01$, *$p < 0.05$.

Figure S3

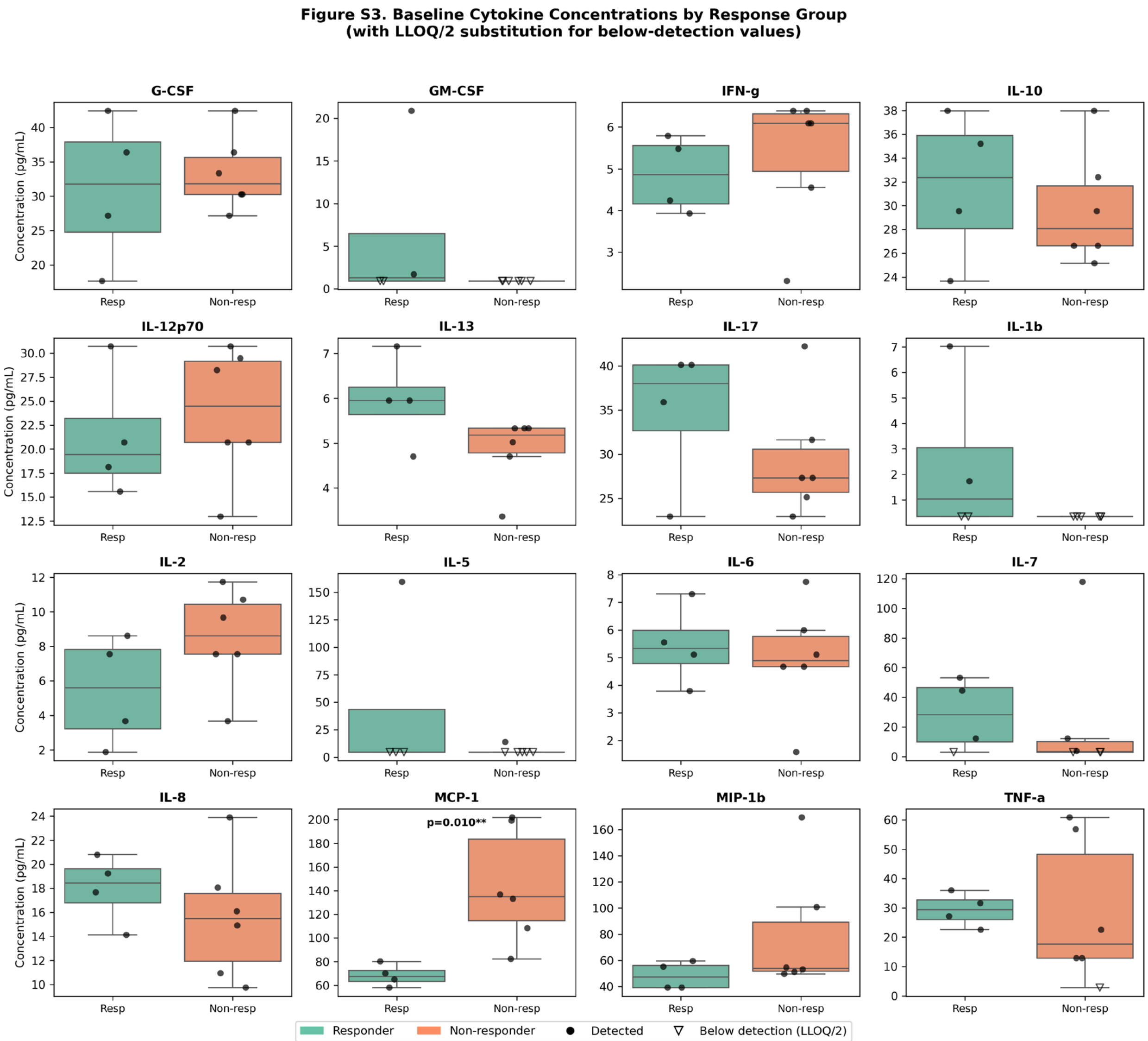

Figure S3. Baseline cytokine concentrations by response group. Comparison of 16 cytokine/chemokine concentrations at baseline between responders (n=4) and non-responders (n=6). One responder (1003-009) lacked baseline cytokine data. Box plots show median with interquartile range. Filled circles indicate detected values; open triangles (▽) indicate values below the assay detection limit, substituted with LLOQ/2 (half the lower limit of quantification). Several cytokines showed high proportions of below-detection values: IL-4 (100%), IL-1β (87%), GM-CSF (85%), IL-5 (79%), IL-7 (46%). Only MCP-1 showed a statistically significant baseline difference between groups, which remained significant after Benjamini-Hochberg correction across the six MCP-1 timepoints (p-adj = 0.036; Figure 2A).

Figure S4

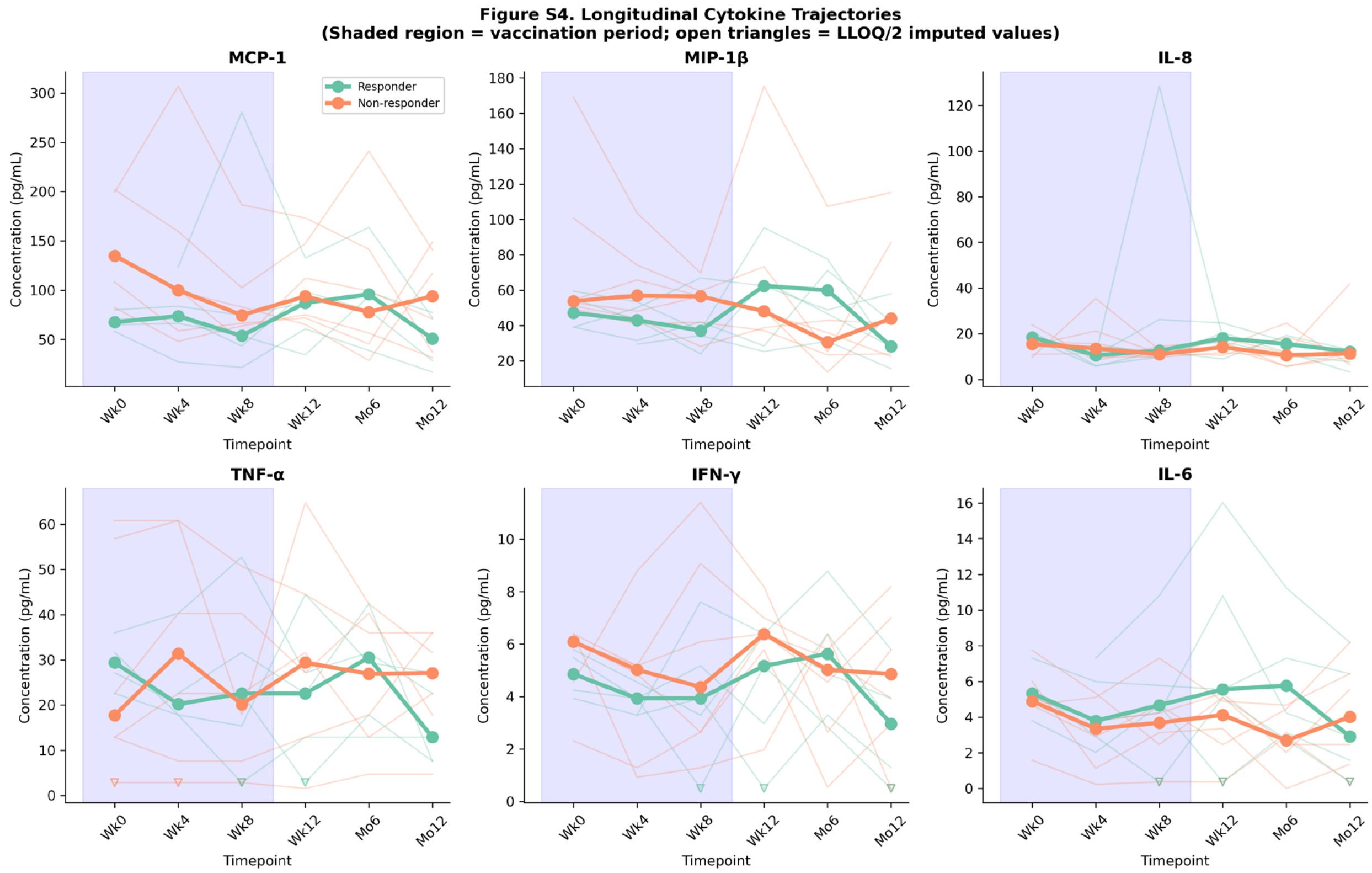

Figure S4. Longitudinal cytokine trajectories. Serum concentrations of key cytokines (MCP-1, MIP-1β, IL-8, TNF-α, IFN-γ, IL-6) across vaccination timepoints. Thin lines represent individual patients, colored by response status (green = responder, orange = non-responder); thick lines with markers show group medians. Open triangles (▽) indicate values below the assay detection limit, substituted with LLOQ/2. Shaded regions indicate vaccination period (Weeks 0–8). n=11 patients at baseline (one responder 1003-009 lacked baseline cytokine data); n=10 at Month 6 (one responder 1002-003 missing sample; one patient 1004-002 lost to follow-up); n=11 at Month 12 (1004-002 lost to follow-up). These six cytokines were selected for visualization based on: (1) consistent detectability above assay limits across samples (below-detection rates: MCP-1, MIP-1β, IL-8 = 0%; TNF-α = 8%; IFN-γ = 6%; IL-6 = 9%), (2) biological relevance as chemokines (MCP-1, MIP-1β), pro-inflammatory markers (IL-6, IL-8, TNF-α), and Th1 cytokines (IFN-γ), and (3) inclusion of the two analytes showing significant associations with response status (MCP-1, MIP-1β; Figure 3). Cytokines with concentrations below detection limit in the majority of samples (IL-4, IL-5, IL-13, GM-CSF, IL-1β) were excluded from this panel.

Figure S5

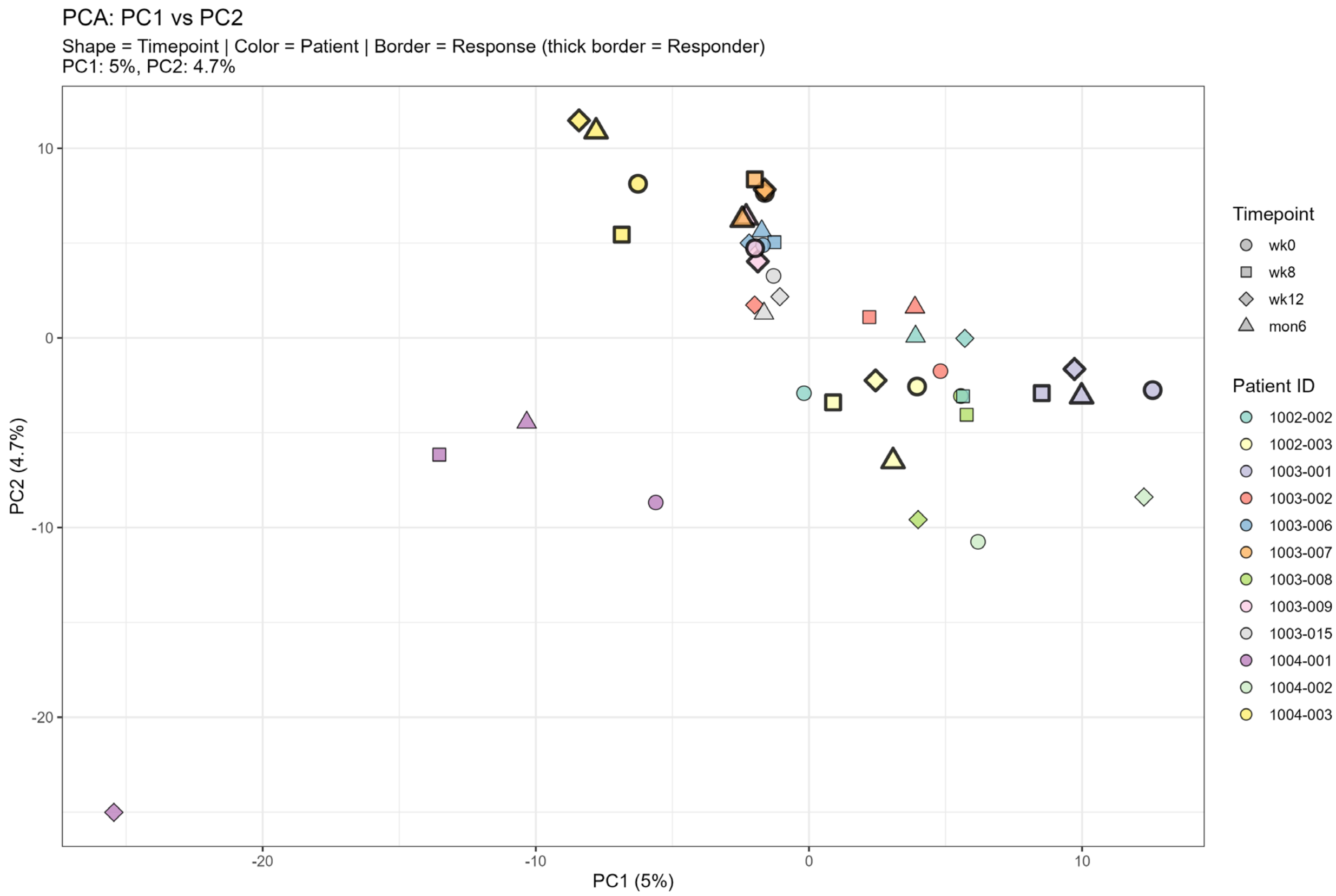

Figure S5. Principal component analysis of TCR repertoire composition by treatment response. PCA ordination of TCRβ repertoire data based on normalized clonotype frequencies. Each point represents one sample (n=43 samples from 12 patients), with fill color encoding patient identity (screening number, shown in the color legend) and shape indicating timepoint (circle = Week 0, square = Week 8, diamond = Week 12, triangle = Month 6). Border thickness indicates treatment response status (thick = responder, thin = non-responder). Individual samples are not annotated with in-plot text labels; patient identity is read from the color legend. PC1 explains 5% and PC2 explains 4.7% of total variance. Notable outlier: Patient 1004-001 at Week 12 (lower left) exhibited a dramatic oligoclonal expansion (Shannon diversity 9.6 vs. 30–120 at other timepoints; clonality 0.19 vs. 0.08–0.09), reflecting a transient dominance by a small number of highly expanded clones that resolved by Month 6.

Figure S6

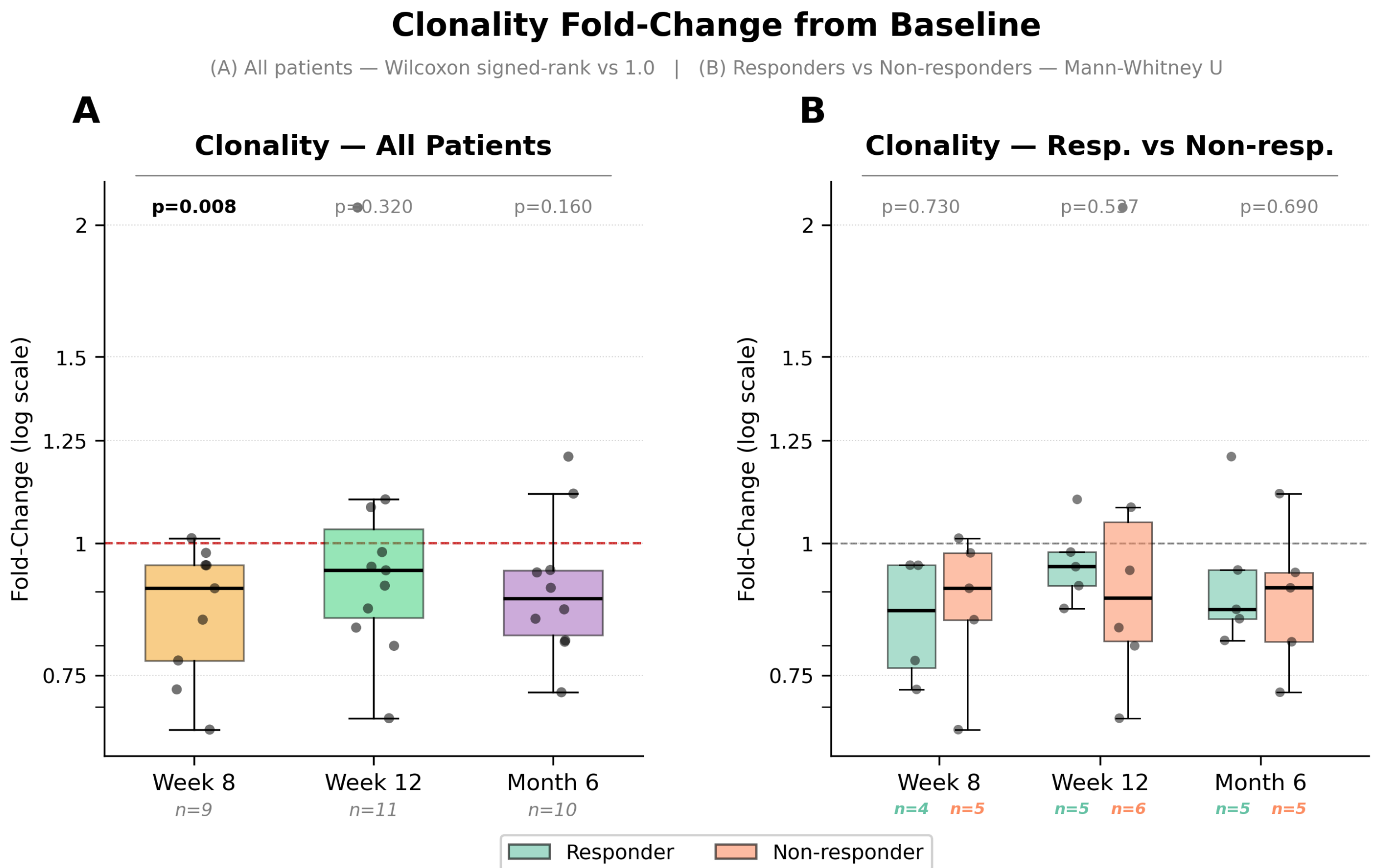

Figure S6. Clonality fold-change from baseline. (A) Clonality fold-change relative to baseline (Week 0) for all patients at post-vaccination timepoints (Week 8, Week 12, Month 6). Clonality is defined as 1 − normalized Shannon entropy; higher values indicate more oligoclonal repertoires. Fold-change > 1 indicates increased clonality (more oligoclonal); fold-change < 1 indicates decreased clonality (more polyclonal). Red dashed line indicates no change from baseline. Box plots show median with interquartile range; individual patient data points are overlaid. Wilcoxon signed-rank test p-values (vs 1.0) shown above each timepoint. Clonality was significantly decreased at Week 8 (p = 0.008), indicating more polyclonal repertoires early after vaccination; subsequent timepoints were not significant. (B) Clonality fold-change compared between responders (green) and non-responders (orange) at each post-vaccination timepoint. Mann-Whitney U test p-values shown above each comparison. No significant differences between groups at any timepoint. n=30 post-baseline observations from 11 patients (5 responders, 6 non-responders; patient 1004-002 excluded). Sample sizes vary by timepoint due to missing samples.

Figure S7

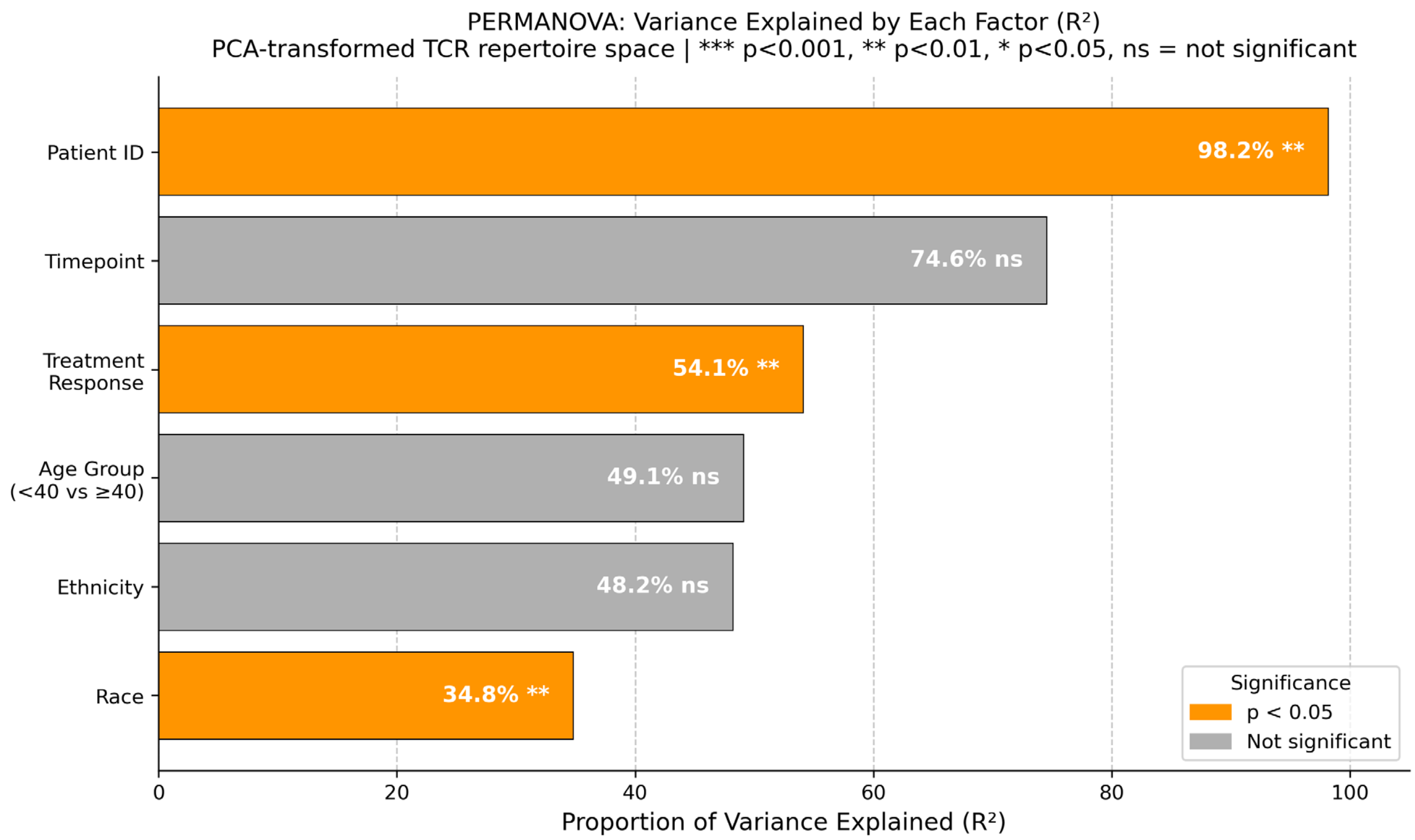

Figure S7. PERMANOVA variance explained by clinical and demographic factors. Horizontal bar plot showing the proportion of variance (R^2) in TCR repertoire composition explained by each factor tested in PERMANOVA analysis of PCA-transformed repertoire space. Factors tested: Patient ID (individual variation), Timepoint (vaccination phase), Treatment Response (HPV16 clearance), Age Group (<40 vs. ≥40 years), Ethnicity (Hispanic vs. non-Hispanic), and Race. Orange bars indicate statistically significant factors ($p < 0.05$); gray bars indicate non-significant factors. Patient ID explained the most variance (98.2%, $p = 0.001$), reflecting the highly individualized nature of TCR repertoires. Treatment Response was the strongest biologically meaningful factor (54.1%, $p = 0.005$). Timepoint was not significant (74.6%, $p = 0.998$) despite high R^2, indicating that within-patient repertoire stability exceeds between-group differences over time. Race significance (34.8%, $p = 0.002$) is likely driven by the single Asian patient (1004-001). Analysis based on 999 permutations. n=43 samples from 12 patients. ***p < 0.001, **p < 0.01, *p < 0.05, ns = not significant.

Figure S8

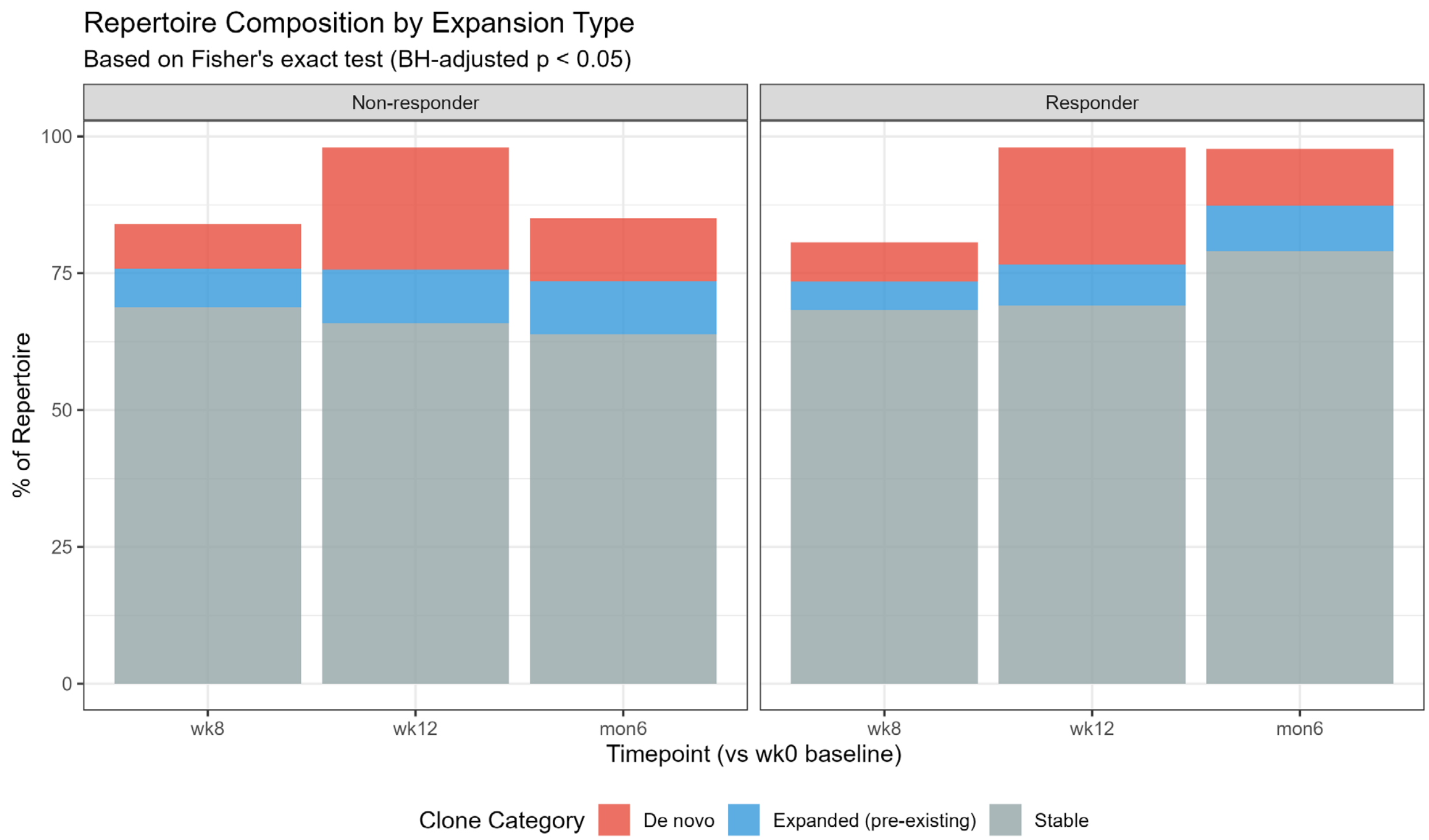

Figure S8. Repertoire composition by expansion type. Fraction of post-vaccination TCR repertoire occupied by different expansion categories: de novo clones (absent at baseline, significantly expanded by Fisher's exact test with BH-adjusted $p < 0.05$), expanded pre-existing clones (present at baseline with significant expansion), and stable clones (no significant change). Stacked bar plots show repertoire fractions at each post-vaccination timepoint (Week 8, Week 12, Month 6), faceted by treatment response (responders vs. non-responders). De novo clones (red) comprised a substantial portion of the post-vaccination repertoire in both groups, representing newly recruited T cell responses. Expanded pre-existing clones (blue) likely represent memory T cells amplified by vaccination. No significant differences in composition were observed between response groups. n=29 patient-timepoints from 11 patients.

Figure S9

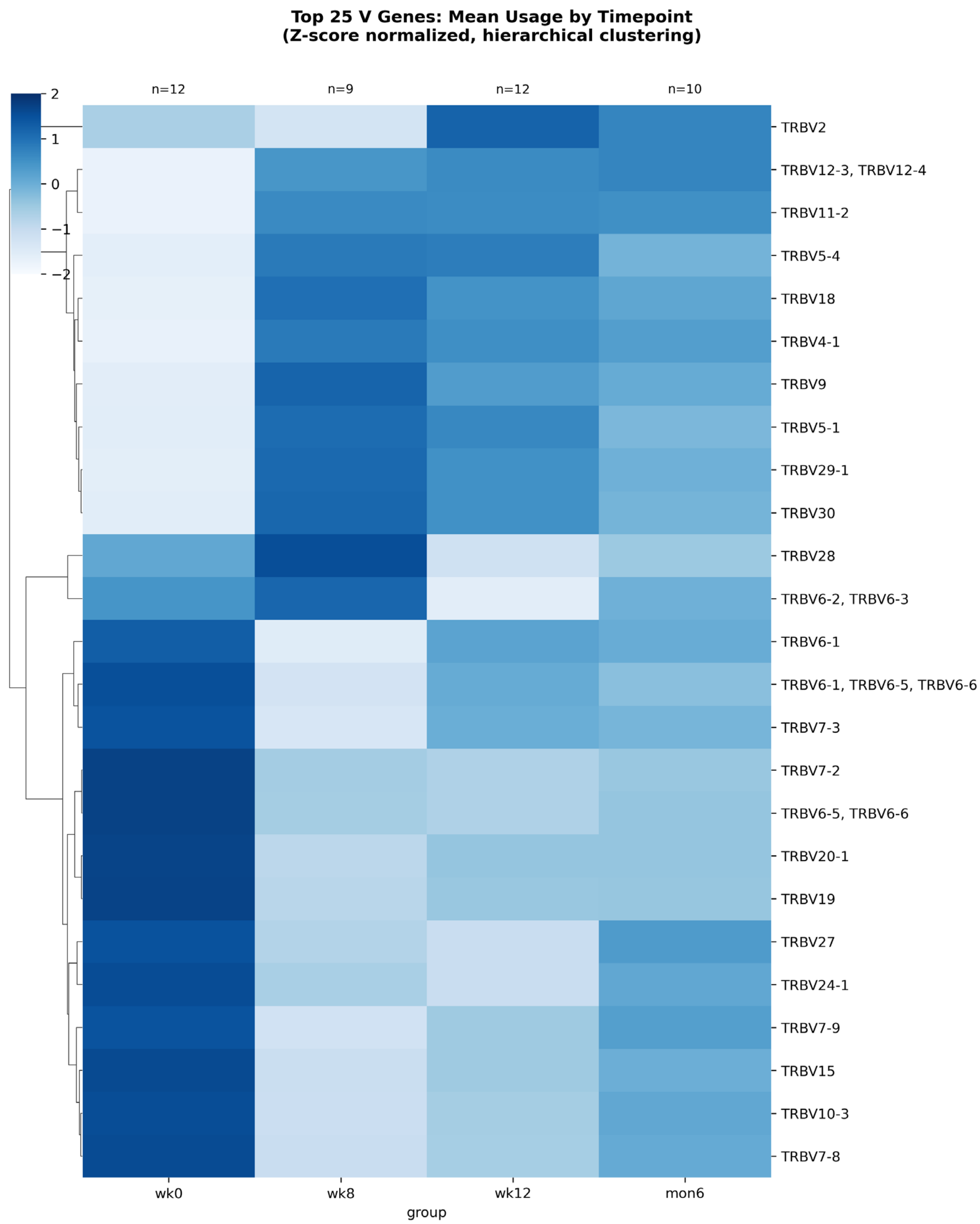

Figure S9. Top 25 TRBV genes across vaccination timepoints. Heatmap showing z-score normalized usage of the 25 most abundant TRBV genes, with mean frequencies calculated per timepoint (Week 0, Week 8, Week 12, Month 6) and z-score transformed across timepoints (row-wise) to highlight relative temporal patterns independent of absolute abundance. Genes are organized by hierarchical clustering (Euclidean distance, complete linkage) with dendrogram shown at left, grouping genes with similar temporal dynamics. Color scale ranges from −2 (blue, relatively lower usage) to +2 (dark blue, relatively higher usage). V gene usage patterns were relatively stable across timepoints, with only TRBV18 (p = 0.048) and TRBV15 (p = 0.007) showing significant temporal changes (Kruskal-Wallis test). n=43 samples from 12 patients (n=12 at Week 0, n=9 at Week 8, n=12 at Week 12, n=10 at Month 6).

Figure S10

A

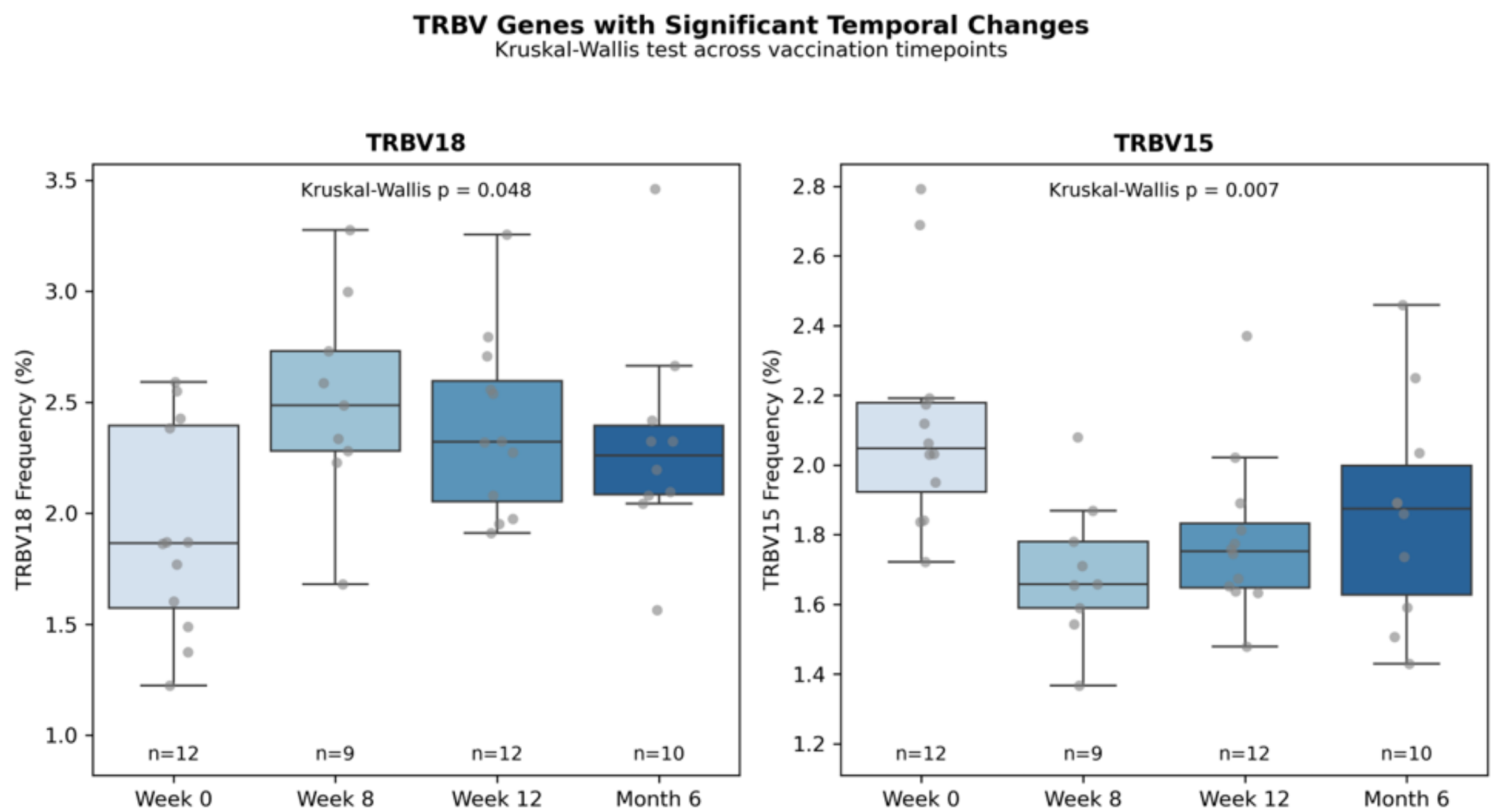

B

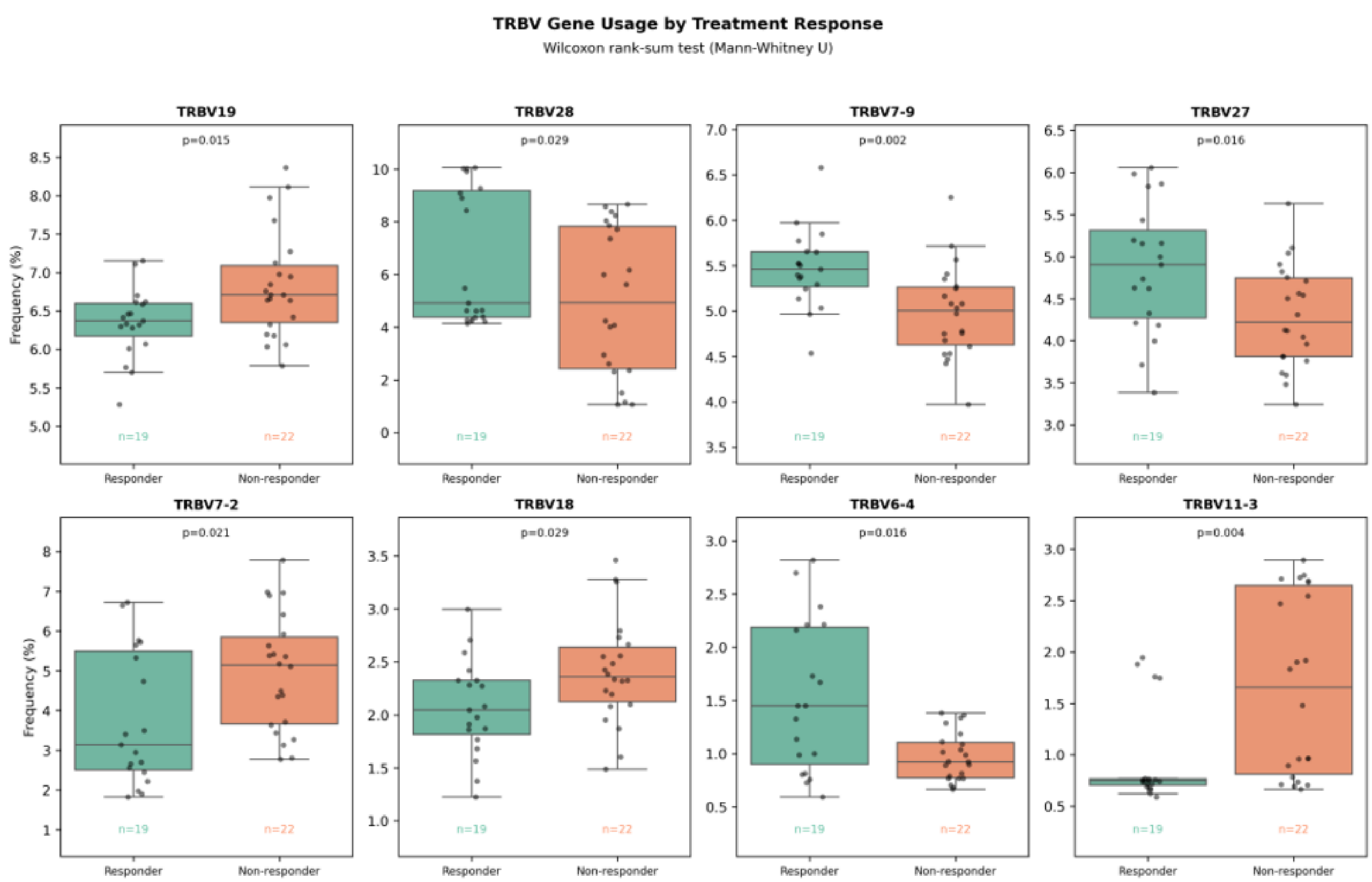

Figure S10. TRBV gene usage: temporal changes and differential usage by treatment response. (A) Box plots showing TRBV18 and TRBV15 usage across vaccination timepoints (Week 0, Week 8, Week 12, Month 6). These were the only V genes showing significant temporal changes: TRBV18 (p = 0.048) and TRBV15 (p = 0.007, Kruskal-Wallis test). n=43 samples from 12 patients (n=12 at Week 0, n=9 at Week 8, n=12 at Week 12, n=10 at Month 6). (B) Box plots comparing the frequency of TRBV genes showing notable differences between responders and non-responders (Mann-Whitney U test). Key differentially used genes include TRBV19, TRBV28, TRBV7-9, TRBV27, TRBV7-2, TRBV18, TRBV6-4, and TRBV11-3. TRBV6-4 and TRBV28 were enriched in responders, while TRBV11-3 and TRBV7-2 showed higher usage in non-responders. n=41 samples from 11 patients (19 responder samples, 22 non-responder samples; patient 1004-002 excluded due to loss to follow-up). For both panels: box plots show median with interquartile range; individual data points overlaid; sample sizes indicated below each box.

Figure S11

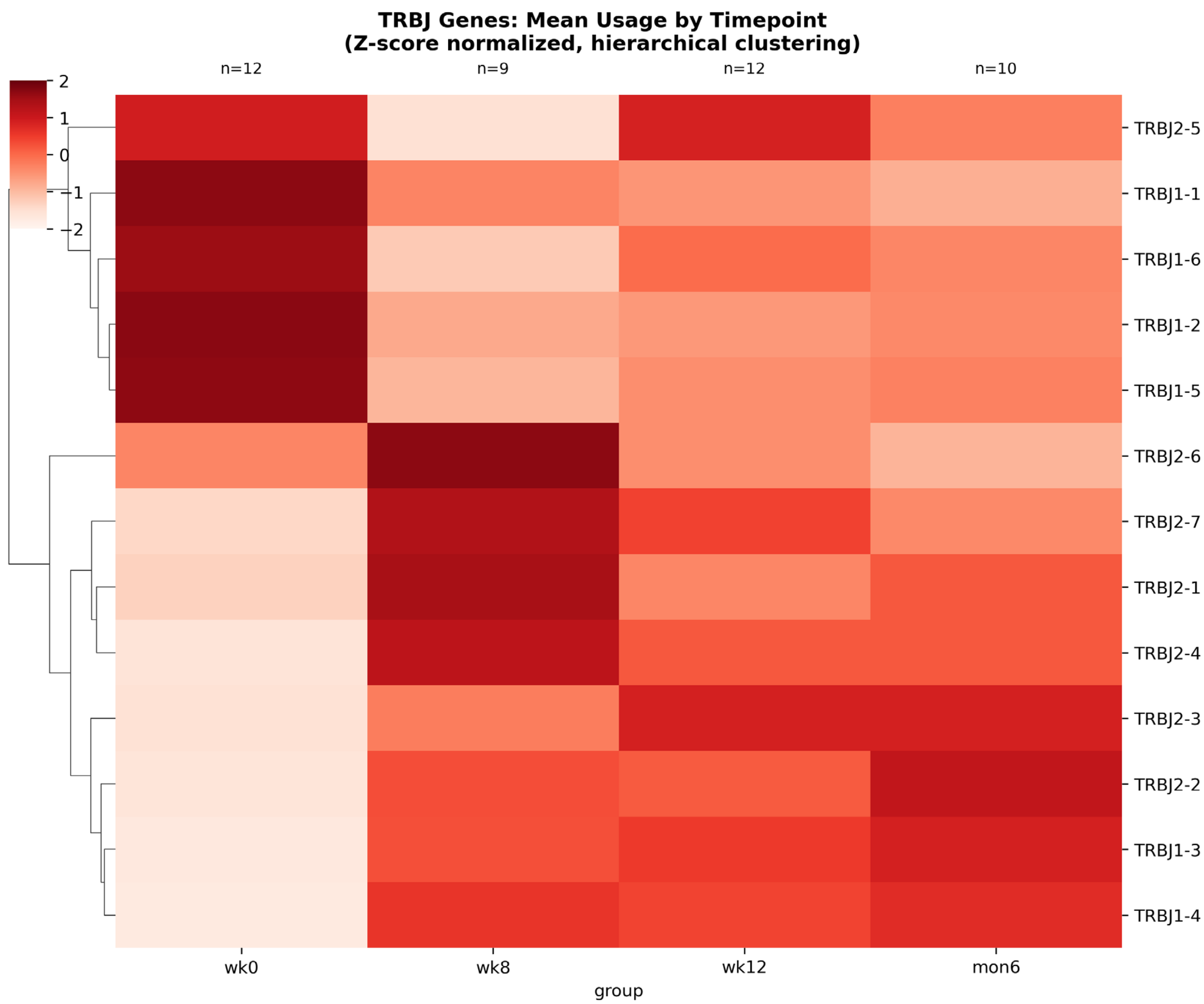

Figure S11. TRBJ gene usage heatmap across vaccination timepoints. Heatmap showing z-score normalized usage of all TRBJ genes, with mean frequencies calculated per timepoint (Week 0, Week 8, Week 12, Month 6) and z-score transformed across timepoints (row-wise) to highlight relative temporal patterns independent of absolute abundance. Genes are organized by hierarchical clustering (Euclidean distance, complete linkage) with dendrogram shown at left, grouping genes with similar temporal dynamics. Color scale ranges from −2 (light pink, relatively lower usage) to +2 (dark red, relatively higher usage). TRBJ2-7 and TRBJ1-1 were consistently the most abundant J genes across all timepoints in

absolute terms. n=43 samples from 12 patients (n=12 at Week 0, n=9 at Week 8, n=12 at Week 12, n=10 at Month 6).

Figure S12

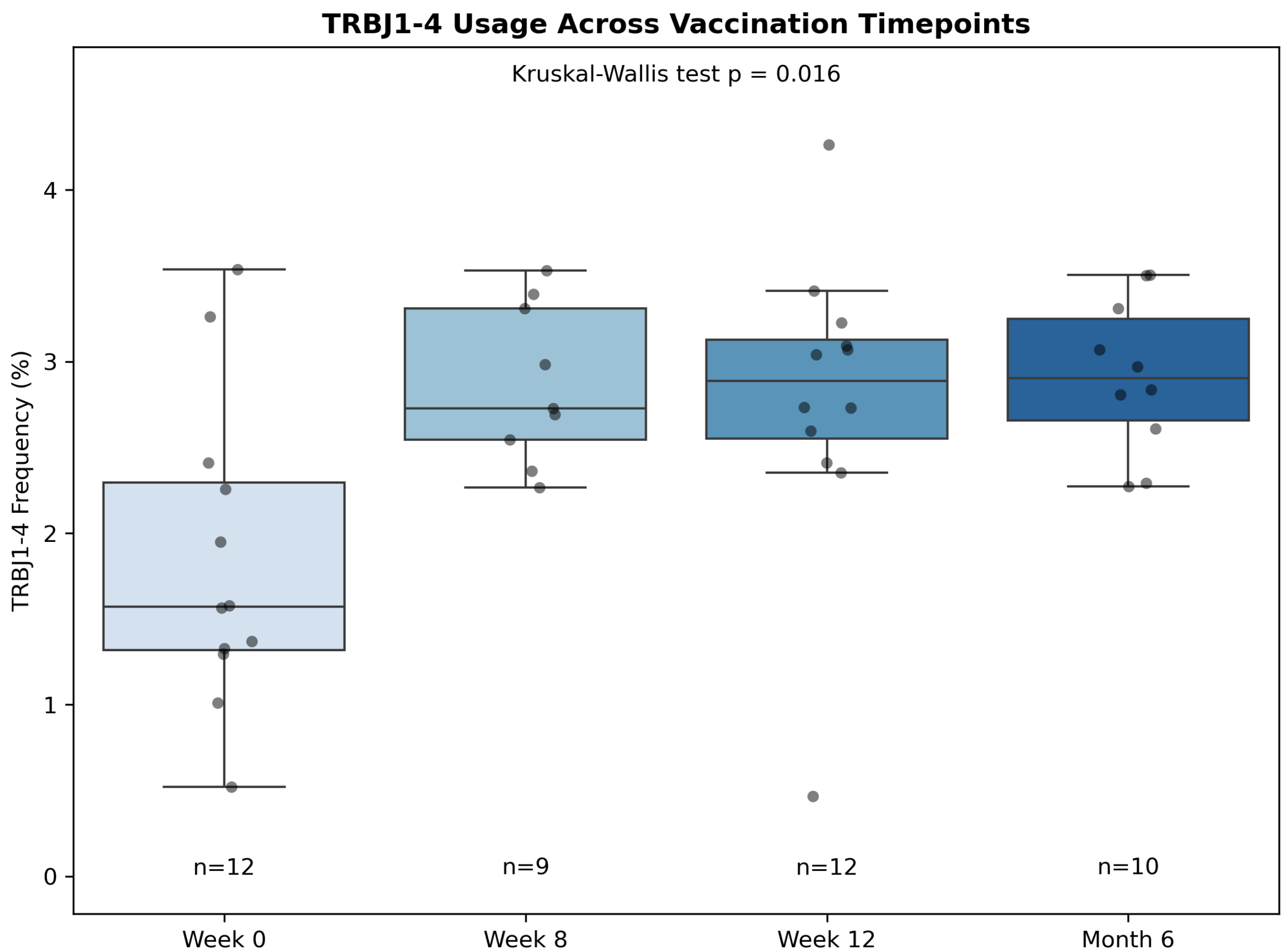

Figure S12. TRBJ genes with significant temporal changes. Box plots showing TRBJ1-4 usage across vaccination timepoints. TRBJ1-4 demonstrated a significant increase from baseline (Week 0) to later timepoints (p = 0.016, Kruskal-Wallis test). Individual data points overlaid on box plots show median with interquartile range. n=43 samples from 12 patients (n=12 at Week 0, n=9 at Week 8, n=12 at Week 12, n=10 at Month 6).

Figure S13

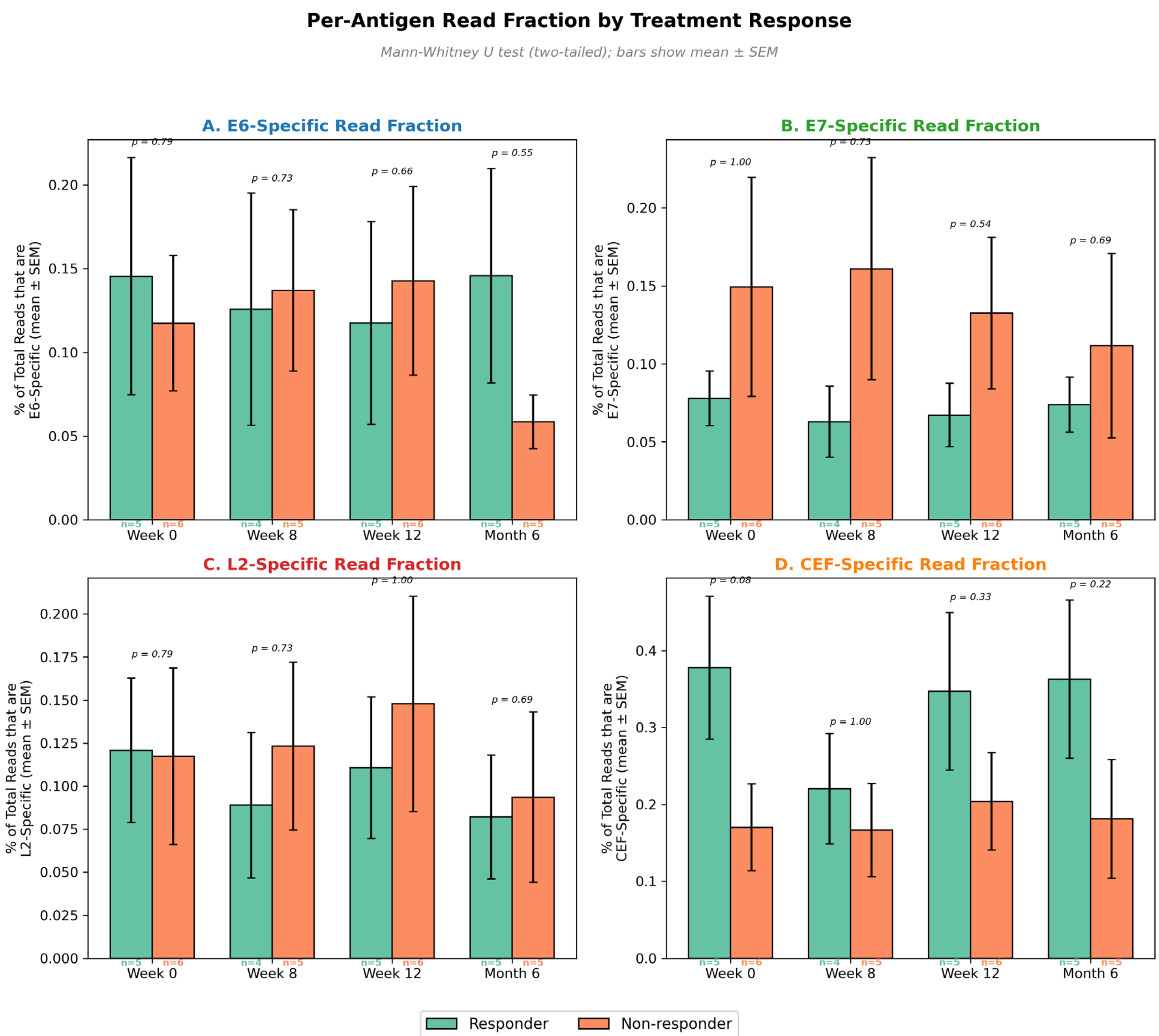

Figure S13. Antigen-specific read fraction by response group across all genomic clones (reads-based analysis). Each panel shows the mean (± SEM) percentage of total sequencing reads attributed to antigen-specific clones in responders (green) versus non-responders (orange) at each timepoint (Week 0, Week 8, Week 12, Month 6). (A) E6-specific read fraction. (B) E7-specific read fraction. (C) L2-specific read fraction. (D) CEF-specific (positive control) read fraction. All panels use identical formatting; n = 5 responders, n = 6 non-responders; patient 1004-002 excluded due to loss to follow-up.

Figure S14

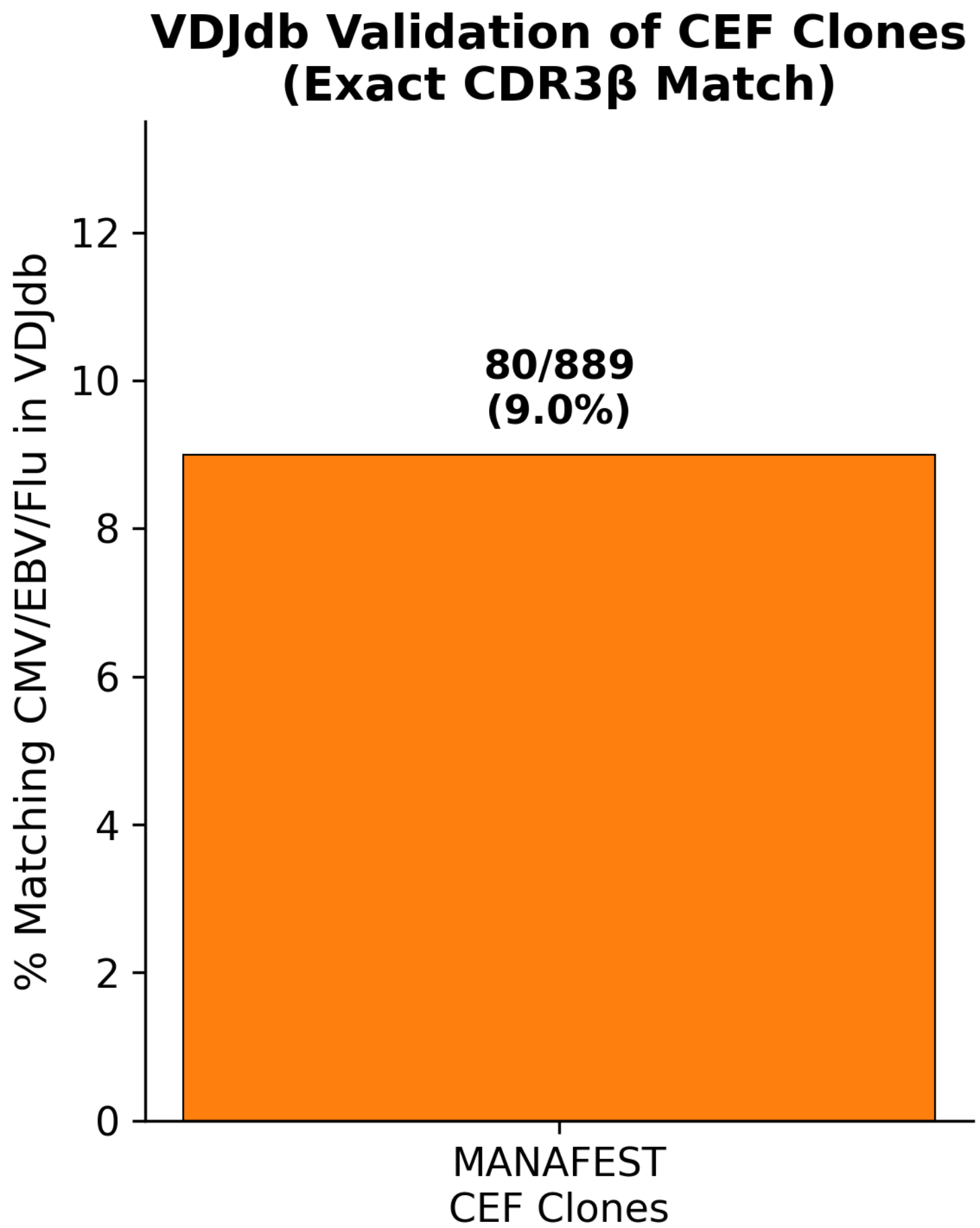

Figure S14. Validation of MANAFEST CEF clone specificity using VDJdb. Percentage of MANAFEST-identified CEF clones versus non-CEF clones in the full genomic repertoire matching CMV/EBV/influenza CDR3β sequences in VDJdb (exact amino acid match). MANAFEST CEF clones showed significant enrichment for VDJdb matches (9.0%, 80/889) compared to non-CEF genomic clones (0.5%, 5,894/1,119,619; Fisher's exact test, one-sided; OR=18.7, p=3.58×10^{-69}). Bar labels indicate the number of matching clones out of total clones and the corresponding percentage. This validation demonstrates that the MANAFEST assay reliably identifies antigen-specific T cell clones, supporting the specificity of HPV antigen assignments used in this study.

Supplementary Methods

HPV16 E6, E7 and L2 Antibody Response Assessment by ELISA

Serum IgG antibodies against HPV16 E6, E7, and L2 proteins were measured by enzyme-linked immunosorbent assay (ELISA). The recombinant HPV16 proteins were purified from bacteria and coated onto 384-well Maxisorp plates at 25 ng per well in 25 µL of Dulbecco's phosphate-buffered saline (DPBS) and incubated overnight at 4°C. Following overnight incubation, plates were washed once with wash buffer, then blocked with 50 µL of blocking buffer (1× DPBS containing 4% skim milk [Difco, BD catalog #232100] and 0.2% Tween 20) for 1.5 hours at 4°C. Patient serum samples were serially diluted 3-fold in blocking buffer across 12 points, starting at 1:20 and ending at 1:3,542,940. Diluted samples (25 µL per well) were incubated for 1 hour at room temperature on a plate shaker at 300 RPM. Plates were washed

three times with wash buffer, then bound antibodies were detected using horseradish peroxidase (HRP)-conjugated goat anti-human IgG (all isotypes; Sigma-Aldrich, catalog #A8667) diluted 1:5000 in blocking buffer (25 µL per well). Following a 1-hour incubation at room temperature on a plate shaker at 300 RPM and three washes with wash buffer, 3,3',5,5'-tetramethylbenzidine (TMB) substrate (TMB 2-Component Microwell Peroxidase Substrate Kit; SeraCare, catalog #5120-0047) was added (25 µL per well) and incubated for 30 minutes at room temperature in the dark. Color development was stopped by adding 2N H_2SO_4 (25 µL per well). Absorbance was measured at 450 nm with background subtraction at 620 nm.

Antibody titers were quantified as the half-maximal effective concentration (EC_{50}) using four-parameter logistic (4PL) regression. Curve fitting was performed in R (version 4.5.0) (R Core Team 2025) using the drc package (Ritz et al. 2015). The 4PL model was defined as:

$$OD = Bottom + \frac{Top - Bottom}{1 + \left(\frac{Dilution}{EC_{50}}\right)^{HillSlope}}$$

where *Top* and *Bottom* represent the upper and lower asymptotes, EC_{50} is the dilution yielding 50% of the maximal response, and *HillSlope* describes the curve steepness. To ensure consistent curve fitting across samples, the upper asymptote (Top) was fixed at 2.52 OD_{450} and the lower asymptote (Bottom) was fixed at 0.02 OD_{450}, based on observed plateau values at the lowest and highest dilutions, respectively. Quality control criteria required $R^2 > 0.90$ and successful model convergence. Higher EC_{50} values indicate stronger antibody responses. Fold-change in antibody titers was calculated as the ratio of post-vaccination EC_{50} to baseline (Week 0) EC_{50} for patients with available baseline samples; one patient (1003-009) lacked a baseline serum sample and was excluded from fold-change analyses. Samples from 12 patients (204 total samples) were analyzed across available timepoints, with 100% model convergence. To validate that detected signals represented true antigen-specific binding rather than non-specific background, no-antigen control samples were analyzed using patient 1004-002 serum tested against buffer-coated wells lacking HPV16 antigen (Figure S1). Temporal changes in antibody titers were assessed using the Wilcoxon signed-rank test comparing fold-change values to 1.0 (no change from baseline). Comparisons between responders and non-responders were performed using the Mann-Whitney U test (two-tailed). Statistical significance was defined as $p < 0.05$. All statistical analyses were performed in R (version 4.5.0).

Serum Cytokine Profiling by Luminex Multiplex Assay

Serum cytokine and chemokine concentrations were measured using the Bio-Plex Pro Human Cytokine 17-plex Assay (Bio-Rad Laboratories, catalog #M5000031YV) on a Bio-Plex 200 Suspension Array System. The panel quantified the following 17 analytes: granulocyte colony-stimulating factor (G-CSF), granulocyte-macrophage colony-stimulating factor (GM-CSF), interferon-gamma (IFN-γ), interleukin-1 beta (IL-1β), IL-2, IL-4, IL-5, IL-6, IL-7, IL-8, IL-10, IL-12(p70), IL-13, IL-17A, monocyte chemoattractant protein-1 (MCP-1/MCAF), macrophage inflammatory protein-1 beta (MIP-1β), and tumor necrosis factor-alpha (TNF-α). The assay employs a sandwich immunoassay format on fluorescently dyed magnetic microspheres. Capture antibodies directed against each cytokine are covalently coupled to beads with distinct spectral addresses, permitting simultaneous detection of multiple analytes. Biotinylated detection antibodies and streptavidin-phycoerythrin (SA-PE) reporter conjugate complete the detection complex. Fluorescence intensity, proportional to analyte concentration, was measured using dual-laser flow cytometry (635 nm for bead classification, 532 nm for PE reporter signal). Serum samples were analyzed across six timepoints: baseline (Week 0), Week 4, Week 8, Week 12, Month 6, and Month 12. A total of 68 samples from 12 patients were available, with sample availability varying by timepoint due to specimen recovery (patient 1004-002 missing Month 6 and Month 12 due to loss to follow-up; patient 1003-009 missing baseline; patient 1002-003 missing Month 6). Prior to analysis, serum samples were centrifuged at 1,000 × g for 15 min at 4°C to remove particulates, then diluted 1:4 in sample diluent HB according to manufacturer's instructions. Diluted samples (50 µL per well, equivalent to 12.5 µL undiluted serum) were tested as single replicates; standards, controls, and blanks were run in duplicate. Standard curves were generated using 4-fold serial dilutions of recombinant cytokine standards provided with the kit, and concentrations were calculated using 5-parameter logistic (5PL) curve fitting. Data were acquired on a

Bio-Plex 200 System using Bio-Plex Manager Software (Bio-Rad) with a minimum of 50 beads counted per analyte per well, and reported as observed concentrations (pg/mL) interpolated from standard curves.

Cytokine concentrations were compared between response groups using the Mann-Whitney U test. Comparisons were made at each individual timepoint (raw concentrations) and as change from baseline to assess vaccination-induced dynamics. Patients were grouped as HPV16 responders (cleared at Month 6, n=5) versus non-responders (persistent at Month 6, n=6). One patient (1004-002) was lost to follow-up and excluded from response comparisons. For samples with concentrations below the assay detection limit (reported as “Out of Range Below” by Bio-Plex Manager), values were imputed as LLOQ/2 (half the lower limit of quantification) to enable statistical comparisons. Several cytokines had high proportions of below-detection values: IL-4 (100%), IL-1β (87%), GM-CSF (85%), IL-5 (79%), IL-7 (46%). Sample sizes for individual comparisons varied due to missing baseline data for patient 1003-009 (Figure S3, S4).

T Cell Receptor Sequencing from Peripheral Blood

T cell receptor beta chain (TCRβ) repertoires were profiled from peripheral blood mononuclear cells (PBMCs) collected at four timepoints: baseline (Week 0), Week 8, Week 12, and Month 6. Genomic DNA was extracted from cryopreserved PBMCs using the QIAamp DNA Micro Kit (QIAGEN, catalog #56304) according to manufacturer’s instructions. TCRβ libraries were prepared and sequenced by the Johns Hopkins FEST and TCR Immunogenomics Core Facility (FTIC) using the AmpliSeq for Illumina TCR beta-SR (Short Read) panel, which employs multiplex PCR with primers targeting all functional TRBV and TRBJ gene segments to amplify the CDR3 region, which is a the hypervariable loop that determines antigen specificity. A total of 43 samples were successfully sequenced from 12 patients. Sample availability varied by timepoint: Week 0 (n=12), Week 8 (n=9), Week 12 (n=12), and Month 6 (n=10), with missing samples due to insufficient PBMC recovery or DNA quality. Libraries were sequenced on an Illumina MiSeq instrument using 151×2 paired-end sequencing. Raw reads were processed using fastp (v0.23) for adapter trimming (Illumina adapter: CTGTCTCTTATACACATCT) and quality filtering (minimum base quality Q30, minimum read length 50 bp). Quality metrics were assessed using FastQC and aggregated with MultiQC.

V(D)J gene annotation and clonotype assembly were performed using MiXCR (v4.3.2) (Bolotin et al. 2015) with the ampliseq-tcrb-sr-cdr3 preset optimized for AmpliSeq short-read TCRβ libraries. MiXCR aligns reads to the human TCRβ reference (IMGT database), assembles overlapping reads spanning the CDR3 region, corrects for PCR and sequencing errors, and outputs clonotype tables containing CDR3 nucleotide and amino acid sequences, V/D/J gene assignments, and read counts.

Non-CDR3 alignments were retained for gene usage analysis. Quality reports were generated for both alignment and assembly steps. Clonotypes were defined by unique CDR3 amino acid sequences.

Repertoire Analysis

Downstream TCR repertoire analysis was performed in R (v4.5.0) (R Core Team 2025) using the immunarch package (v0.9.0) (Nazarov et al. 2025). All analyses were conducted on clonotypes defined by unique CDR3 amino acid sequences.

Sequencing Depth Normalization

For diversity analyses, rarefaction was performed by subsampling all samples to the minimum sequencing depth observed across the cohort. This normalization ensures that diversity metrics (Shannon, Simpson, Chao1, D50, Clonality) are not confounded by differences in sequencing effort and allows for unbiased cross-sample comparisons. Other analyses (clonal expansion, gene usage, k-mer analysis) used raw read counts to preserve sensitivity for detecting rare expanding clones.

Diversity Metrics

Repertoire diversity was quantified using four complementary indices, each calculated on rarefied data:

Shannon diversity measures both clonotype richness and evenness. Shannon entropy is calculated as:

$$H = -\sum_{i=1}^{S} p_i \ln(p_i)$$

where p_i is the frequency of clonotype i and S is the total number of unique clonotypes. Shannon diversity is reported as the True Diversity $\exp(H)$, representing the effective number of equally abundant clonotypes (analogous to the inverse Simpson index). Higher values indicate more diverse repertoires with balanced clonotype frequencies.

Inverse Simpson index $(1/D)$ represents the effective number of equally abundant clonotypes, calculated as:

$$D = \sum_{i=1}^{S} p_i^2$$

This metric is more sensitive to changes in dominant clones than Shannon entropy.

Chao1 estimator provides an estimate of total clonotype richness including unobserved rare clonotypes, accounting for undersampling of the true repertoire diversity.

D50 index represents the minimum number of clonotypes accounting for 50% of total sequencing reads. Lower D50 values indicate repertoires dominated by fewer expanded clones.

Clonality was calculated as $1 - (H_2/\log_2(N))$, where H_2 is Shannon entropy computed using base-2 logarithms and N is the total number of unique clonotypes. Clonality ranges from 0 (perfectly even repertoire) to 1 (monoclonal), with higher values indicating repertoires dominated by fewer expanded clones. To assess vaccination-induced changes in diversity, fold-change from baseline (Week 0) was calculated for each metric at each post-vaccination timepoint (Week 8, Week 12, Month 6). One-sample Wilcoxon signed-rank tests evaluated whether fold-change differed from 1.0 (no change). Mann-Whitney U tests compared fold-change between responders and non-responders at each timepoint.

Clonal Expansion Analysis

To identify vaccine-induced clonal expansion, we performed longitudinal tracking of individual clonotypes. For each patient with baseline (Week 0) and at least one post-vaccination sample (Week 8, Week 12, or Month 6), we calculated the $\log_2$ fold-change in clonotype frequency:

$$\log_2\text{FC} = \log_2\left(\frac{f_{post} + \epsilon}{f_{baseline} + \epsilon}\right)$$

where f_{post} and $f_{baseline}$ represent clonotype frequencies at post-vaccination and baseline timepoints, respectively, and $\epsilon = 10^{-9}$ is a pseudocount to handle zero frequencies. To determine statistical significance, a one-sided Fisher's exact test was applied to each clonotype's 2×2 contingency table of read counts (clone vs. non-clone reads at baseline vs. post-vaccination), testing whether the clone's proportion increased post-vaccination. P-values were adjusted for multiple testing within each patient using the Benjamini-Hochberg method. Clonotypes were classified as "expanding" if they reached statistical significance (adjusted $p < 0.05$) and the post-vaccination frequency exceeded 0.01%. Clonotypes showing a statistically significant change with decreased fold-change (adjusted $p < 0.05$, fold-change < 1) were classified as "contracting." To quantify the overall magnitude of vaccine-induced expansion per patient, a cumulative expansion score was calculated as the sum of $\log_2$ fold-changes across all expanding clonotypes at each post-vaccination timepoint. Public expanding clones (defined as clonotypes expanding in multiple patients) were identified to assess between-patient convergence of the T cell response. The number of patients sharing each expanding clone and the mean $\log_2$ fold-change were recorded. Responder-specific expanding clones were additionally defined as CDR3 sequences that expanded (adjusted $p < 0.05$) in two or more responders but not in any non-responder, representing candidate HPV16-reactive T cell responses.

V and J Gene Usage

The frequency of each TRBV and TRBJ gene segment was calculated per sample as the proportion of total reads assigned to each gene. Gene frequencies were compared between responders and non-responders using $\log_2$ fold-change analysis:

$$\log_2\text{FC} = \log_2\left(\frac{\bar{f}_{responders}}{\bar{f}_{non-responders}}\right)$$

where $\bar{f}$ represents the mean gene frequency within each group. Positive values indicate enrichment in responders; negative values indicate enrichment in non-responders. Temporal changes in gene usage were assessed using Kruskal-Wallis tests across the four timepoints. For visualization of temporal patterns, mean gene frequencies per timepoint were z-score normalized across timepoints (row-wise) to highlight relative changes independent of absolute abundance, and genes were organized by hierarchical clustering (Euclidean distance, complete linkage) to group genes with similar temporal dynamics. V-J gene pairing frequencies were calculated and visualized as hierarchically clustered heatmaps to identify preferential gene combinations.

Dimensionality Reduction and Multivariate Analysis

To assess overall repertoire composition differences, principal component analysis (PCA) was performed on clonotype frequency matrices. Multidimensional scaling (MDS) was applied to Jaccard distance matrices to visualize sample relationships in reduced dimensions. To test whether repertoire composition significantly differed by clinical or demographic factors, permutational multivariate analysis of variance (PERMANOVA) was performed using the adonis2 function from the vegan R package (999 permutations) on PCA-transformed clonotype frequency data. We tested the following factors: patient identity (to quantify individual variation), treatment response, vaccination timepoint, age group (<40 vs. ≥40 years), race, and ethnicity. Each factor was tested independently to assess its contribution to repertoire variation.

Public Clonotype Identification

T cells recognizing the same antigen can independently generate identical CDR3 sequences in different individuals, a phenomenon known as “public” or “convergent” TCR responses. The presence of shared clonotypes across multiple patients provides strong evidence of a common antigen-driven response. To identify such convergent vaccine responses, public clonotypes (CDR3 sequences shared across multiple patients) were identified and their frequency tracked across timepoints. The number of patients sharing each public clonotype was calculated. To quantify the extent of between-patient repertoire convergence, we computed the mean percentage of each patient’s unique expanding clonotypes that were shared with other patients at the same timepoint. Pairwise differences between timepoints were assessed using Mann-Whitney U tests with Bonferroni correction for three comparisons, and responder versus non-responder comparisons at each timepoint were performed using the Mann-Whitney U test. Shared expanding clones were cross-referenced with the MANAFEST peptide-reactive clone list to determine antigen specificity.

Peptide-Stimulation TCR Sequencing (MANAFEST Assay)

To identify antigen-specific T cell clonotypes, peptide-stimulation TCR sequencing was performed using the MANAFEST (Mutation-Associated NeoAntigen Functional Expansion of Specific T cells) approach (Danilova et al. 2018). Cryopreserved PBMCs from each patient were pooled from available vaccination and follow-up timepoints (4–7 vials per patient, median 6; spanning 2–4 unique timepoints, median 3) to maximize cell recovery and ensure sufficient material for 13 parallel peptide stimulations. The specific timepoints pooled varied by specimen availability; nearly all patients’ pooled samples were exclusively post-vaccination (Week 4 through Month 12), with only Patient 1004-002 contributing a baseline (Week 0) sample. No patient contributed PBMCs from all six collection timepoints. While pooling prevents assessment of temporal dynamics in peptide-specific responses, it provides a cumulative snapshot of each patient’s HPV-reactive T cell repertoire accumulated throughout the vaccination course. Pooled cells were stimulated in vitro with overlapping 15-mer peptide pools (11 amino acid overlap) representing:

- **HPV16 E6**: Three pools (E6A, E6B, E6C) spanning the full E6 protein
- **HPV16 E7**: Three pools (E7A, E7B, E7C) spanning the full E7 protein
- **HPV16 L2**: Three pools (L2A, L2B, L2C) spanning the full L2 protein
- **Positive control**: CEFx peptide pool (CMV, EBV, influenza epitopes)
- **Negative control**: HIV peptide pool (irrelevant specificity)
- **Unstimulated control**: NP (no peptide)

On Day 0, thawed PBMCs were sorted into T cell and non-T cell populations using the EasySep Human T Cell Enrichment Kit (STEMCELL Technologies, catalog 1003-008051). T cells and non-T cells were combined at a 1:1 ratio (5×10^5 total cells) and distributed equally across 13 conditions in 96-well plates ($\sim 3.8 \times 10^4$ cells per well) in 200 µL of IMDM supplemented with 5% human serum (Sigma-Aldrich, catalog #H3667) and 50 µg/mL gentamicin. Stimulating peptides were added at a final concentration of 5 µg/mL. On Day 3, half of the culture medium was replaced with fresh medium containing IL-2 (100 IU/mL), IL-7 (50 ng/mL), and IL-15 (50 ng/mL). On Day 7, a second half-medium exchange was performed with fresh medium containing IL-2 (200 IU/mL), IL-7 (50 ng/mL), and IL-15 (50 ng/mL). On Day 10, cultured cells were harvested from each well. Genomic DNA was extracted from the harvested cells using the QIAamp DNA Micro Kit (QIAGEN, catalog #56304), and TCRβ libraries were prepared and sequenced by the Johns Hopkins FEST and TCR Immunogenomics Core Facility (FTIC) using the AmpliSeq for Illumina TCR beta-SR panel. Samples were pooled and sequenced on an Illumina MiSeq. This analysis generated samples from 13 patients across 13 peptide conditions, including technical replicates for one patient (1003-008 and 1003-008r) to assess assay reproducibility.

Statistical Analysis of MANAFEST Data

MANAFEST data were analyzed using the replicateFest R package (Danilova and Cope 2024). For each patient, clonotype read counts were aggregated by CDR3 amino acid sequence from productive rearrangements only (excluding sequences containing stop codons or frameshift indicators). Clones were required to have ≥50 reads in at least one peptide condition to be considered for analysis, ensuring adequate statistical power. Peptide-enriched clonotypes were identified using negative binomial regression, which models overdispersed count data and accounts for replicate structure. For each clone, a generalized linear model (GLM) with a negative binomial distribution was fit to the read counts across peptide conditions, with clone identity and peptide condition as covariates (model: counts ~ clone × peptide). Odds ratios (OR) were derived from the model coefficients, comparing each peptide-stimulated condition to the unstimulated no-peptide control (NP). P-values were adjusted for multiple testing using the Benjamini-Hochberg method to control the false discovery rate (FDR). Clonotypes were classified as significantly enriched in a peptide condition if they met all of the following criteria: (1) FDR-adjusted p-value < 0.05 and (2) OR ≥ 1. To identify clones uniquely responsive to a single antigen, a uniqueness filter was applied: clones were required to show statistically significant enrichment in only one peptide condition (the “best” condition) and to be significantly different from both the second-best and third-best conditions. This was implemented by comparing the best-responding condition to the second-best and third-best conditions using additional statistical tests, requiring OR ≥ 1 and FDR < 0.05 for both comparisons.

Using these criteria, antigen-specific clones were classified by peptide category. HPV-specific clones were defined as clonotypes that: (1) showed unique significant enrichment in at least one HPV peptide condition (E6, E7, or L2), and (2) did NOT meet enrichment criteria in the HIV negative control condition (to exclude non-specific responders). Clones showing enrichment in both HPV and HIV conditions were excluded as potentially cross-reactive or non-specifically expanded. Each HPV-specific clone was assigned to its single best-responding peptide category (E6, E7, or L2) based on the highest OR value. CEF-reactive clones were similarly identified as clonotypes showing unique significant enrichment in the CEFx condition, serving as a positive control for assay validation. Public peptide-responsive clones were defined as CDR3 sequences enriched in the same peptide condition across multiple patients.

Cross-Reference of Peripheral Blood TCR Repertoire with MANAFEST Antigen Specificity

To determine whether clones detected in peripheral blood were specific for HPV antigens, we cross-referenced all clonotypes from longitudinal bulk TCRseq with the antigen-specific clonotypes identified by MANAFEST. Cross-reference matching was performed by CDR3 amino acid sequence within the same patient: a clone was classified as antigen-specific if its CDR3 sequence was found in that patient's MANAFEST antigen-specific clone set (E6, E7, L2, or CEF). To account for differences in sequencing depth between patients, all comparisons were performed using fraction-based metrics rather than absolute clone counts. Specifically, the antigen-specific fraction was calculated as the percentage of total sequencing reads attributable to antigen-specific clones for each patient at each timepoint. Per-patient, per-timepoint fractions were compared between responders and non-responders using the Mann-Whitney U test (two-tailed). Analyses were performed both for combined HPV antigens (E6 + E7 + L2) and for each antigen individually (E6, E7, L2, CEF).

In Silico Validation of MANAFEST Specificity

To validate the specificity of the MANAFEST assay, CEF-reactive clones (identified as significantly enriched in the CEFx peptide condition) were cross-referenced against VDJdb (Bagaev et al. 2020), a curated database of TCR sequences with known antigen specificity, filtering for CMV, EBV, and influenza A/B entries. The proportion of MANAFEST-identified CEF clones with exact CDR3β amino acid matches to CMV/EBV/influenza entries in VDJdb was compared to the proportion among non-CEF clones using Fisher's exact test (one-sided, testing for enrichment in CEF clones).

References

Bagaev, Dmitry V., Renske M. A. Vrber, Jessica Samir, Ulrik Stervbo, Timm Kunber, Hugo Barcenilla, Remy Stark, Sónia Pinto, Dmitriy M. Chudakov, and Mikhail Shugay. 2020. "VDJdb in 2019: Database Extension, New Analysis Infrastructure and a T-cell Receptor Motif Compendium." *Nucleic Acids Research* 48 (D1): D1057–62. https://doi.org/10.1093/nar/gkz874.

Bolotin, Dmitriy A., Stanislav Poslavsky, Igor Mitrophanov, Mikhail Shugay, Ilgar Z. Mamedov, Ekaterina V. Putintseva, and Dmitriy M. Chudakov. 2015. "MiXCR: Software for Comprehensive Adaptive Immunity Profiling." *Nature Methods* 12 (5): 380–81. https://doi.org/10.1038/nmeth.3364.

Danilova, Ludmila, Valsamo Anagnostou, Justina X. Caushi, John-William Sidhom, Haidan Guo, Hok Yee Chan, Prerna Suri, et al. 2018. "The Mutation-Associated Neoantigen Functional Expansion of Specific T-Cells (MANAFEST) Assay: A Sensitive Platform for Monitoring Antitumor Immunity." *Clinical Cancer Research* 24 (24): 6129–40. https://doi.org/10.1158/1078-0432.CCR-18-0129.

Danilova, Ludmila, and Leslie Cope. 2024. *replicateFest: Analysis of Peptide-Stimulation TCR Sequencing with Replicates*. https://github.com/OncologyQS/replicateFest.

Nazarov, V., V. Tsvetkov, A. Popov, and I. Balashov. 2025. *immunarch: Multi-Modal Immune Repertoire Analytics for Immunotherapy and Vaccine Design in R*. https://github.com/immunomind/immunarch.

R Core Team. 2025. "R: A Language and Environment for Statistical Computing." Vienna, Austria: R Foundation for Statistical Computing. https://www.R-project.org/.

Ritz, Christian, Florent Baty, Jens C. Streibig, and Daniel Gerhard. 2015. "Dose-Response Analysis Using R." *PLOS ONE* 10 (12): e0146021. https://doi.org/10.1371/journal.pone.0146021.
